# Transcriptional integration of mitogenic and mechanical signals by Myc and YAP

**DOI:** 10.1101/gad.301184.117

**Published:** 2017-10-15

**Authors:** Ottavio Croci, Serena De Fazio, Francesca Biagioni, Elisa Donato, Marieta Caganova, Laura Curti, Mirko Doni, Silvia Sberna, Deborah Aldeghi, Chiara Biancotto, Alessandro Verrecchia, Daniela Olivero, Bruno Amati, Stefano Campaner

**Affiliations:** 1 Center for Genomic Science of IIT@SEMM (Istituto Italiano di Tecnologia at European School of Molecular Medicine), Fondazione Istituto Italiano di Tecnologia (IIT), 20139 Milan, Italy;; 2 Department of Experimental Oncology, European Institute of Oncology (IEO), 20139 Milan, Italy;; 3 Laboratorio di Analisi Veterinarie BiEsseA, 20129 Milan, Italy

**Keywords:** YAP, TEAD, Myc, transcription, Hippo signaling

## Abstract

Mammalian cells must integrate environmental cues to determine coherent physiological responses. The transcription factors Myc and YAP–TEAD act downstream from mitogenic signals, with the latter responding also to mechanical cues. Here, we show that these factors coordinately regulate genes required for cell proliferation. Activation of Myc led to extensive association with its genomic targets, most of which were prebound by TEAD. At these loci, recruitment of YAP was Myc-dependent and led to full transcriptional activation. This cooperation was critical for cell cycle entry, organ growth, and tumorigenesis. Thus, Myc and YAP–TEAD integrate mitogenic and mechanical cues at the transcriptional level to provide multifactorial control of cell proliferation.

## Abstract (precis)

The transcription factors Myc and YAP–TEAD act downstream from mitogenic signals, with the latter responding also to mechanical cues. Here, Croci et al. show that these factors coordinately regulate genes required for cell proliferation.

Cell cycle entry in higher eukaryotes depends on extracellular cues mediated by growth factors, metabolites, cell adhesion, and cell–cell contacts. These signals must be interpreted and integrated by cells to regulate complex gene expression programs. Mitogenic agents, such as serum growth factors, stimulate cell proliferation by activating waves of transcription, starting with immediate early genes, which in turn regulate the expression of delayed early genes. c-*myc* is an immediate early gene whose product, the transcription factor (TF) Myc, is essential for serum-mediated and growth factor-mediated cell cycle entry (Kelly et al. 1983; Armelin et al. 1984; Roussel et al. 1991; Barone and Courtneidge 1995; de Alboran et al. 2001; Trumpp et al. 2001; Perna et al. 2012). This function of Myc stems from its ability to control the expression of a large fraction of genes involved in cell activation and proliferation.

When ectopically expressed in quiescent cells, Myc is able to drive cell cycle progression in the absence of serum (Eilers et al. 1991; Pelengaris et al. 1999). This effect of Myc is context-dependent, however, since not all cells or tissues respond to Myc by entering the cell cycle (Jackson et al. 1990; Xiao et al. 2001; Murphy et al. 2008). This suggests that a full proliferative response may require the engagement of other TFs, which may respond to different regulatory signals, such as metabolic or mechanical cues. Recently, YAP has emerged as a key TF in the control of cell growth and organ size in response to a variety of signals such as cell adhesion, apico–basolateral polarity, cytoskeletal tension, and mitogens. YAP activity is controlled by a cascade of regulatory kinases, the Hippo pathway, and mechanotransduction: When either Hippo signaling is low or in conditions of high cytoskeletal tension, YAP translocates into the nucleus, where it associates with TEAD TFs to regulate transcription (Vassilev et al. 2001).

Here we show that YAP coadjuvates Myc-dependent transcription and cooperates in inducing cell cycle entry and cell proliferation both in vitro and in vivo. This depends on the constitutive binding of TEAD to a large fraction of Myc target genes. At these promoters, Myc binding was independent of YAP and led to increases in the histone methylation mark H3K4me3. While this was insufficient for full transcriptional activation, it converted these loci into high-affinity binding sites for YAP. Thus, in low Myc conditions, YAP was bound to its canonical targets, was redistributed to a fraction of Myc target genes upon Myc accumulation, and favored activation of those loci. This multilayered circuit explains how Myc can selectively control gene expression despite its extensive genomic interactions (Kress et al. 2015) and how the modulation of Myc transcriptional programs in *cis* by other TFs allows proper integration of diverse mitogenic stimuli.

## Results and Discussion

**Figure 1.**
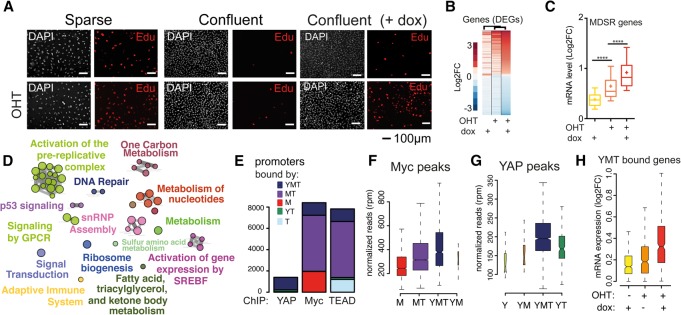
Myc and YAP coregulate cell cycle entry. Serum-starved subconfluent (sparse) (*A*) or highly confluent (confluent) 3T9^MycER;YAP^ (*B*–*H*) cells were treated with OHT to activate MycER and doxycycline (dox) to trigger the expression of YAP^S127A^. (*A*) Cell cycle entry was measured by immunofluorescence analysis of EdU incorporation. DAPI was used to color nuclei. (*B*) Ranked heat map based on the log_2_ fold change of the differentially expressed genes (DEGs) identified by RNA sequencing (RNA-seq). (*C*) Box plot of the mRNA expression level of the Myc-dependent serum response (MDSR) genes (*D*) Gene ontology map based on the DEGs determined upon both MycER activation and YAP induction. (*E*) Cumulative bar graph of Myc, YAP, and TEAD ChIP-seq (chromatin immunoprecipitation [ChIP] combined with high-throughput sequencing) peaks, color-coded based on their overlap. (Y) YAP; (M) Myc; (T) TEAD. (*F*,*G*) Box plot of the enrichment of Myc (*F*) and YAP (*G*) ChIP-seq peaks divided into subsets as in *E*. (*H*) Expression levels of up-regulated genes cobound at their promoters by YAP, Myc, and TEAD (YMT peaks).

To dissect Myc-induced proliferation, we used 3T9^MycER^ fibroblasts, which constitutively express MycER^TM^, a chimeric protein that can be conditionally activated by 4-hydroxytamoxifen (OHT) (Littlewood et al. 1995). As reported (Eilers et al. 1991; Barone and Courtneidge 1995), activation of MycER in serum-starved subconfluent cells was sufficient to stimulate cell cycle entry and Myc-dependent transcription (Fig. 1A; Supplemental Fig. S1A,B). However, these effects were strongly inhibited by cellular confluence (Fig. 1A; Supplemental Fig. S1A,B), suggesting that cell–cell contact and low cytoskeletal tension are inhibitory to Myc-driven proliferation.

While Myc controls cell cycle entry in response to mitogenic signals, YAP also regulates cell proliferation in response to mechanical and cytoskeletal cues (Zhao et al. 2007; Dupont et al. 2011; Fernandez et al. 2011; Sansores-Garcia et al. 2011; Wada et al. 2011; Halder et al. 2012; Aragona et al. 2013). To address whether YAP could cooperate with MycER in cell cycle entry, we transduced 3T9^MycER^ cells with a doxycycline-inducible vector expressing the activated mutant YAP^S127A^ (referred to here as 3T9^MycER;YAP^ cells) (Supplemental Fig. S1C; Zhao et al. 2007). While either MycER or YAP^S127A^ alone had no significant effect in serum-starved confluent cells, their coactivation resulted in robust cell cycle entry (Fig. 1A; Supplemental Fig. S1D,E). This was paralleled by the differential expression of a large number of genes (DEGs [differentially expressed genes]) that responded to MycER and YAP together but not—or less significantly—to either alone (Fig. 1B; Supplemental Fig. S2A–D). These genes were linked mainly to cell proliferation (Fig. 1E; Supplemental Fig. S2E,F) and included previously identified Myc-dependent serum response (MDSR) genes (Fig. 1C; Supplemental Fig. S2D; Perna et al. 2012). In a general manner, the response of MycER-induced genes was augmented by coactivation of YAP (Fig. 1C; Supplemental Fig. S2D).

This was not due to reciprocal regulation of MycER and YAP protein levels or nuclear localization, which were largely unaffected by their coexpression both in vitro and in vivo (Supplemental Figs. S1C, S3A–G). Instead, MycER activation increased the chromatin-associated fraction of YAP (Supplemental Fig. S3H).

Consistent with the above, ChIP-seq (chromatin immunoprecipitation [ChIP] combined with high-throughput sequencing) analysis revealed extensive overlaps in the genomic localization of YAP, TEAD, and Myc (YMT) (Supplemental Fig. S4,A–C). In particular, virtually all YAP-associated promoters were co-occupied by TEAD and Myc (Fig. 1E). Genomic sites cobound by the three proteins (i.e., YMT peaks) showed the greatest enrichment for each single TF (Fig. 1F,G; Supplemental Fig. S4D,E). Genes cobound at their promoters by the three TFs showed stronger transcriptional responses to YAP and Myc together than to either TF alone (Fig. 1H). Thus, YAP and Myc coregulate a subset of Myc target genes, in particular those linked to cell cycle entry.

**Figure 2.**
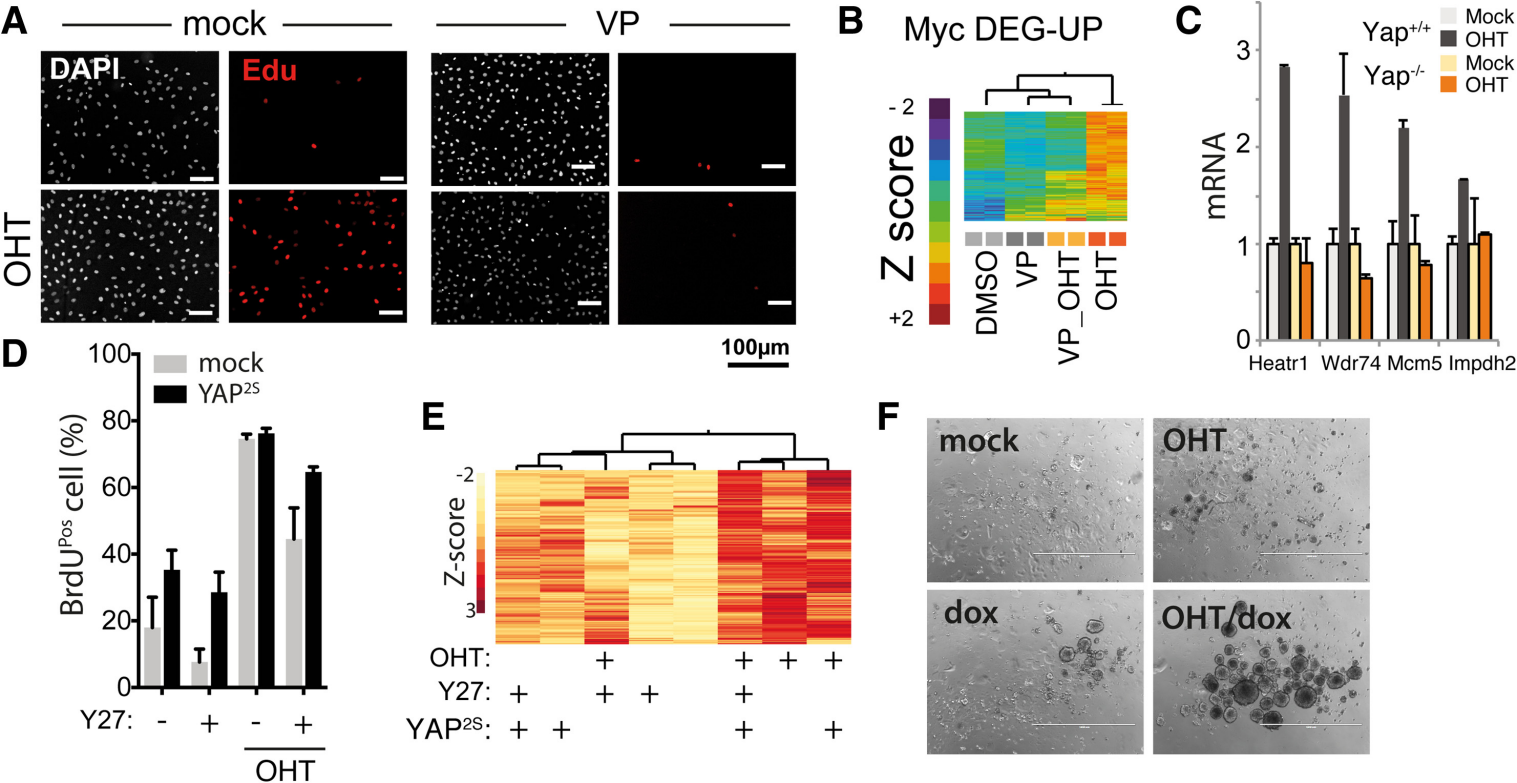
Myc-driven cell cycle entry depends on YAP activity and cytoskeletal tension. (*A*–*E*) Serum-starved subconfluent fibroblasts were kept in low serum and treated as indicated. (*A*) Immunofluorescence analysis of Myc-induced cell cycle entry of 3T9^MycER^ measured as EdU incorporation on cells treated with the YAP inhibitor verteporfin (VP). (*B*) Expression analysis (clustering) of Myc up-regulated genes following VP treatment. (*C*) RT-qPCR expression of Myc target genes in MycER fibroblasts either wild type (*YAP^+/+^*) or knockout (*Yap^−/−^*) for *Yap*. (*D*) S-phase entry by BrdU incorporation (by FACS) in MycER fibroblasts overexpressing YAP^S127A/S318A^. Cells were treated with OHT to activate MycER and with the ROCK inhibitor Y276632 (Y27) as indicated. (*E*) Clustered heat map of normalized mRNA expression of cells shown in *D*. (*F*) Anchorage-independent growth assay of bipotential mouse embryonic liver (BMEL) cells overexpressing MycER and tet-YAP^S127A^, treated as indicated. Representative pictures of cell colonies are shown.

In sparse cultures, where high cytoskeletal tension activates YAP (Zhao et al. 2007; Dupont et al. 2011; Fernandez et al. 2011; Sansores-Garcia et al. 2011; Wada et al. 2011; Halder et al. 2012; Aragona et al. 2013), YAP inhibitors blocked Myc-induced cell cycle entry and transcription of Myc target genes (Fig. 2A,B; Supplemental Figs. S5, S6). Similarly, YAP deletion impaired the expression of Myc target genes (Fig. 2C). In the same conditions, pharmacological relief of cytoskeletal tension (with blebbistatin, an inhibitor of actomyosin contraction) or blockade of actin-mediated signaling (with the ROCK inhibitor Y276632) impaired both Myc-induced cell cycle entry and induction of Myc target genes, which were rescued by overexpression of an activated YAP mutant (YAP^S127/381A^) (Fig. 2D,E; Supplemental Fig. S7). Overall, these data suggest that Myc-induced transcription and cell cycle entry rely on the activation of endogenous YAP by cytoskeletal tension. In line with this, coexpression of YAP^S127A^ promoted anchorage-independent growth of MycER cells (a growth condition where cytoskeletal tension is low) while not providing any proliferative advantage in two-dimensional conditions (Fig. 2F; Supplemental Fig. S8).

**Figure 3.**
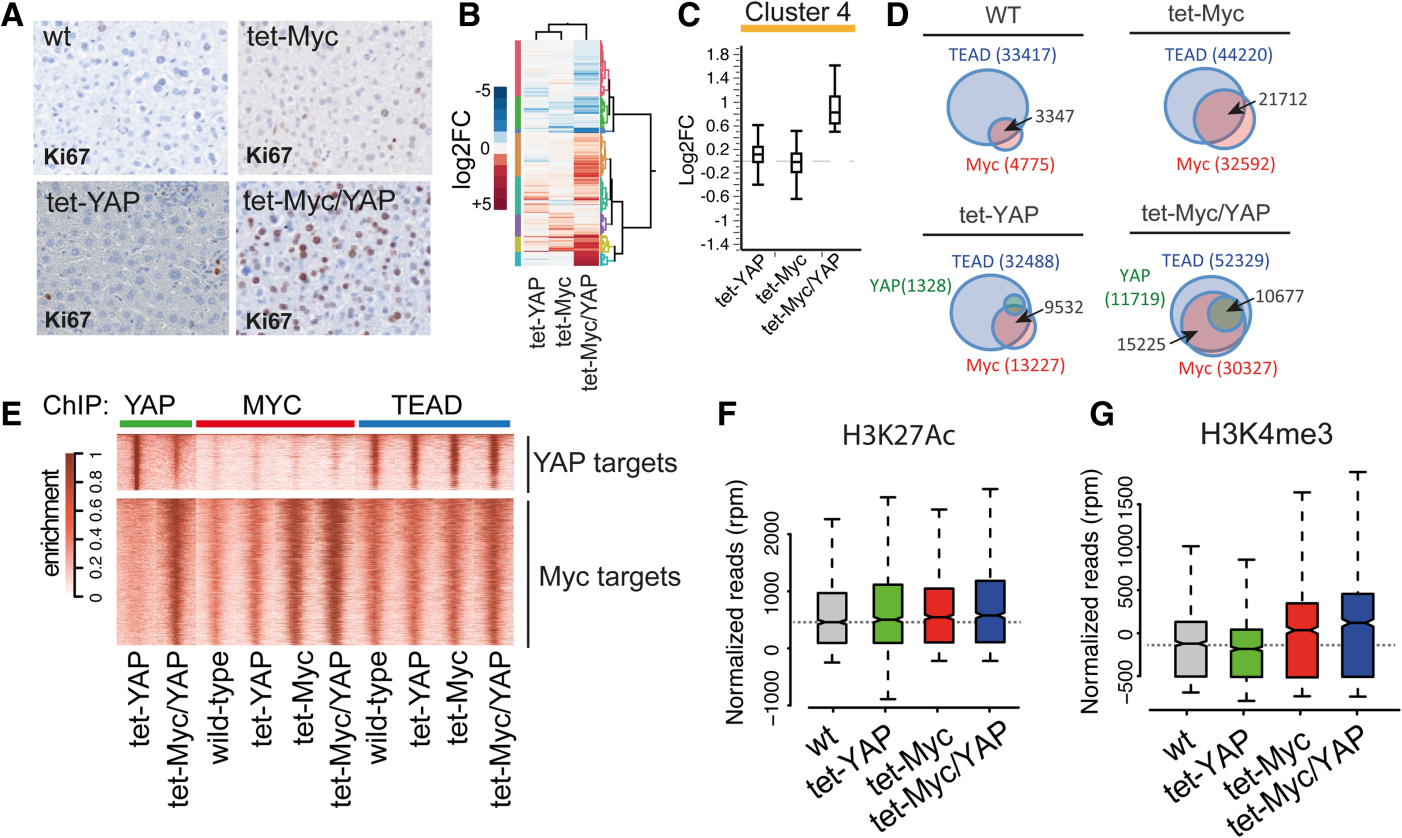
Cooperative binding and transcriptional activation by Myc, TEAD, and YAP. Genome-wide analyses of livers from R26-rtTA mice either wild type (wt), transgenics for Myc (tet-Myc) or YAP (tet-YAP), or double transgenics (tet-Myc/YAP). Short-term induction was achieved by feeding mice with doxycycline-containing food for 48 h. (*A*) Liver sections stained with an anti-Ki67 antibody. (*B*) Hierarchical clustering of DEGs. (*C*) Box plot showing a representative cluster of YAP/Myc DEGs. (*D*) Venn analysis of Myc, YAP, and TEAD ChIP-seq peaks. The number of peaks determined for each TF is reported in brackets; the arrows point to the number of overlapping peaks. (*E*) Ranked heat maps of the ChIP-seq enrichment of the indicated TFs. (*Top* panel) YAP peaks detected only in tet-YAP livers. (*Bottom* panel) Promoters bound by YAP in tet-Myc/YAP livers. (*F*,*G*) H3K27ac (*F*) and H3K4me3 (*G*) levels at promoters of DEG-up genes cobound by Myc and YAP.

We then addressed the relevance of our finding in a post-mitotic adult tissue. Toward this aim, we used mouse strains allowing doxycycline-inducible expression of Myc (tet-Myc) (Beer et al. 2004; Shachaf et al. 2004; Kress et al. 2016) and YAP (tet-YAP) (Jansson and Larsson 2012) in the liver. While short-term induction (48 h) of either Myc or YAP alone led to a mild proliferative response, their coactivation (tet-Myc/YAP) resulted in robust cellular proliferation (Fig. 3A; Supplemental Fig. S9). Genome-wide expression analysis by RNA sequencing (RNA-seq) revealed a large number of genes (2500) coregulated by YAP and Myc but not by either factor alone (Fig. 3B,C; Supplemental Fig. S10A–E). These genes were enriched for ontological terms linked mainly to cell proliferation and included the aforementioned MDSR genes (Supplemental Fig. S11). On the other hand, YAP target genes were down-modulated by coexpression of Myc (cluster 6) (Supplemental Fig. S10E,F), consistent with the reported antagonism between Myc and YAP signaling (von Eyss et al. 2015).

We then profiled the genomic interactions of Myc, YAP, and TEAD by ChIP-seq in the liver. While Myc displayed limited chromatin association in wild-type hepatocytes (only 5000 peaks), its short-term induction led to extensive chromatin binding (>30,000 peaks). A comparable increase of Myc peaks was detected in tet-Myc/YAP livers, indicating that Myc binding to chromatin depended on its expression level but not on YAP expression (Fig. 3D; Supplemental Fig. S12A). TEAD showed widespread chromatin interactions with a similar genomic distribution among the four experimental groups (Fig. 3D; Supplemental Fig. S12A). YAP showed no significant chromatin association in either wild-type or tet-Myc mice, consistent with its low expression level (Supplemental Figs. S3F, S12A). However, YAP peaks became detectable in tet-YAP and were further boosted in tet-Myc/YAP mice in both number and level of enrichment (Fig. 3D,E; Supplemental Fig. S12A,B). Importantly, in tet-YAP livers, YAP showed only a partial genomic overlap with Myc, while coexpression in tet-Myc/YAP favored its recruitment to genomic sites bound by both Myc and TEAD (Fig. 3D,E). Thus, Myc caused a global shift in the genomic distribution of YAP, favoring its recruitment to promoters bound by both Myc and TEAD (Fig. 3E, bottom panel; Supplemental Fig. S12C) and its concomitant decrease from the canonical TEAD/YAP targets most significantly enriched in the livers of tet-YAP mice (Fig. 3E, top panel). This reshuffling of YAP away from its canonical binding sites provides a rationale for the repression of YAP target genes that we and others (von Eyss et al. 2015) observed upon Myc activation.

In tet-Myc/YAP mice, genomic loci cobound by YMT peaks showed stronger enrichment of all TFs compared with regions bound by each TF alone (Supplemental Fig. S12D). Likewise, genomic loci cobound by YMT in tet-Myc/YAP livers had the strongest enrichment for each TF when both Myc and YAP were overexpressed compared with all other conditions (Fig. 3E; Supplemental Fig. S12E). Importantly, a consistent number of genes differentially expressed in tet-Myc/YAP cells were cobound at their promoters by YMT (Supplemental Fig. S12F).

Myc generally requires a pre-existing active chromatin environment to access DNA, characterized in particular by the histone marks H3K4me3 and H3K27ac (Guccione et al. 2006; Sabo et al. 2014; Kress et al. 2015); indeed, in wild-type livers, these marks pre-existed on the promoters of Myc/YAP coregulated genes (Supplemental Fig. S12G,H). At these loci, H3K27ac was slightly increased by binding of YAP, Myc, or both (Supplemental Fig. S12G). H3K4me3 was low in wild type and tet-YAP but increased upon Myc binding and was further enhanced by YAP, implying a role of TF-induced chromatin modifications in stabilizing the YMT complex on these sites (Fig. 3F; Supplemental Fig. S12H; Stein et al. 2015). This suggested cooperative binding of these TFs, with Myc and TEAD favoring YAP recruitment to their common target loci.

**Figure 4.**
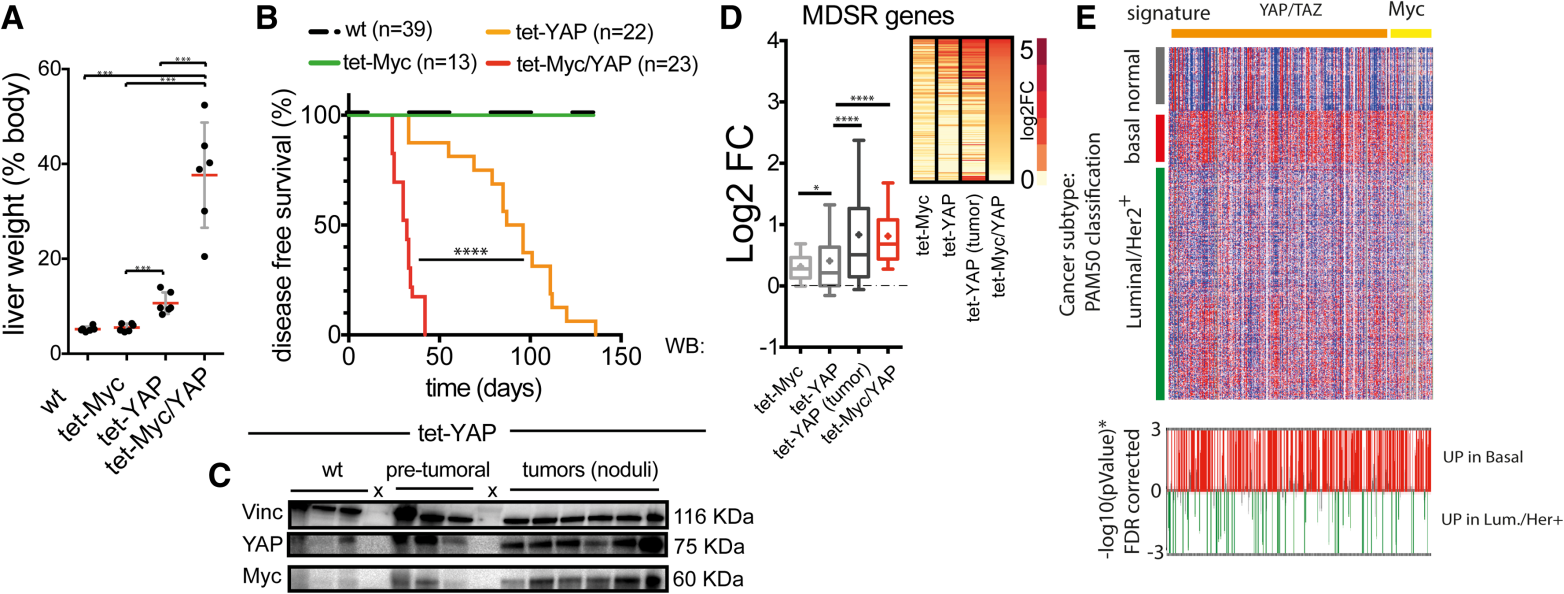
Myc and YAP cooperate in inducing liver growth and tumorigenesis. (*A*) Liver weight assessed at 5 wk of induction. Data are reported as percentage relative to total body weight. (*B*) Kaplan-Meier disease-free survival analysis. (*C*) Western blotting analysis of YAP and Myc levels in LAP-tTA tet-YAP mice at the pretumoral stage (4 wk of YAP activation) and in tumors. Vinculin (vin) was used as aninternal control for equal loading. (*D*) Box plot of the expression level of MDSR genes up-regulated in the liver upon YAP and/or Myc induction. (*Inset* at the *right*) Ranked heat map. (*E*, *top* panel) Heat map of Myc and YAP/TAZ gene signatures based on the expression data of breast cancers (TCGA_BRCA). The heat map was clustered by breast cancer subtypes (basal-like, normal-like, and Luminal/Her2^+^). (*Bottom* panel) The statistical track shows the logarithmic plot of *P*-values for each gene. (Red bars) Genes up in basal-like; (green bars) genes up in Luminal/Her^+^.

Next, we wondered whether induction of Myc and YAP might also have long-term consequences on liver growth. As reported (Camargo et al. 2007; Dong et al. 2007), tet-YAP mice developed mild hepatomegaly within 5 wk of induction (Fig. 4A), indicating that sustained activation of YAP in the liver can result in a proliferative response that is likely due to the engagement of secondary cellular programs (Camargo et al. 2007; Yimlamai et al. 2014). tet-Myc mice showed no signs of liver enlargement. Instead, coinduction of Myc and YAP led to massive hepatomegaly, which accounted for the remarkably short disease-free survival of these mice (Fig. 4A,B; Supplemental Fig. S13A). Histologically, these livers were classified as hepatocellular carcinomas with a diffuse solid pattern of growth, indicating pervasive aberrant proliferation (Supplemental Fig. S13B–D).

As reported (Camargo et al. 2007; Dong et al. 2007), prolonged activation of YAP alone led to the development of focal tumor lesions with full penetrance. This was paralleled by a progressive elevation of Myc levels, which peaked in tumoral lesions (Fig. 4C,D; Supplemental Fig. S14A–C). MDSR genes were progressively up-regulated to reach levels in tumors that were comparable with those observed in tet-Myc/YAP mice (Fig. 4D). RNA-seq analysis showed similarities among YAP-driven tumors and tet-Myc/YAP livers, both of which clustered apart from tet-Myc or tet-YAP alone (Supplemental Fig. S14D). These data suggest a selective pressure for the up-regulation of Myc in YAP-driven tumors, reiterating the strong mitogenic effect observed upon coactivation of both oncogenes. Accordingly, the Myc and Hippo gene signatures were coenriched in basal-like breast tumors (Fig. 4E), a subset with reported deregulation of the Hippo pathway (Cordenonsi et al. 2011), and both the YAP/TAZ and Myc signatures could independently stratify basal-like tumors (Supplemental Fig. S15).

In summary, we described here a *cis*-regulatory network comprising Myc and the YAP–TEAD complex that controls the expression of proliferative genes. The promoters of genes activated by Myc and YAP were prebound by TEAD, were heavily bookmarked by chromatin activation marks, and showed stalled RNA polymerase II (Supplemental Fig. S12I), indicating that they were poised for activation. Myc binding was YAP-independent, although the presence of YAP favored further stabilization of Myc on chromatin. On the other hand, recruitment of YAP to Myc–TEAD-bound promoters was fully dependent on prebound Myc. Our data suggest that YAP recruitment on Myc sites is favored by Myc-induced epigenetic remodeling (which increases H3K4me3 and, to a lower extent, H3K27ac levels) as well as protein–protein interaction between Myc and YAP (Supplemental Fig. S16). Further work will be needed to fully dissect the molecular mechanism of YAP recruitment to these loci.

Altogether, our findings provide a parsimonious solution for the integration of mitogenic and mechanical signals in the control of cell cycle entry and proliferation. Our data also illustrate how convergent signals (here, YAP activation) endow Myc with the ability to control selective transcriptional responses in spite of its pervasive association with the genome (Perna et al. 2012; Sabo et al. 2014; Walz et al. 2014; Kress et al. 2015; Piccolo et al. 2017).

## Materials and methods

### Mouse strains

Tet-YAP mice (*Col1A1-YAP^S127A^* transgenic mice) were kindly provided by Dr. Jonas Larsson. tet-MYC transgenic mice were a kind gift from Dr. Martin Eilers. For liver-specific transgene expression, mice were crossed with LAP-tTA mice expressing the tTA tetracycline transactivator under the control of the LAP promoter [B6.Cg-Tg(*tTALap*)5Bjd/J; purchased from Jackson Laboratories].

### Cell culture

3T9^MycER^ murine fibroblasts (Sabo et al. 2014) were infected with pSlik-YAP^S127A^ retroviruses and selected with 100 µg/mL hygromycin. MycER was activated by the addition of 20–400 nM OHT, while YAP^S127A^ was induced by 2 µg/mL doxycycline. Detailed experimental procedures and data analysis are in the Supplemental Material.

RNA-seq and ChIP-seq data have been deposited in NCBI's Gene Expression Omnibus (GEO) and are accessible through GEO series accession number GSE83869.

## Supplementary Material

Supplemental Material
