# Supplementary material for: Transcriptional integration of mitogenic and mechanical signals by Myc and YAP
Source: Genes Dev. 2017 Oct 15;31(20):2017–22. doi: 10.1101/gad.301184.117 (PMC5733494; doi:10.1101/gad.301184.117)
Supplement: Supplemental Material [file supp_31_20_2017__index.html]

Transcriptional integration of mitogenic and mechanical signals by Myc and YAP — Supplemental Material 

# Transcriptional integration of mitogenic and mechanical signals by Myc and YAP

## Supplemental Material

- Supplemental\_material.pdf
- Supplemental\_data\_Croci.xlsx
