# Supplementary material for: Transcriptional integration of mitogenic and mechanical signals by Myc and YAP
Source: Genes Dev. 2017 Oct 15;31(20):2017–22. doi: 10.1101/gad.301184.117 (PMC5733494; doi:10.1101/gad.301184.117)
Supplement: Supplemental Material [file supp_gad.301184.117_Supplemental_material.pdf]

Supplemental Figure S1

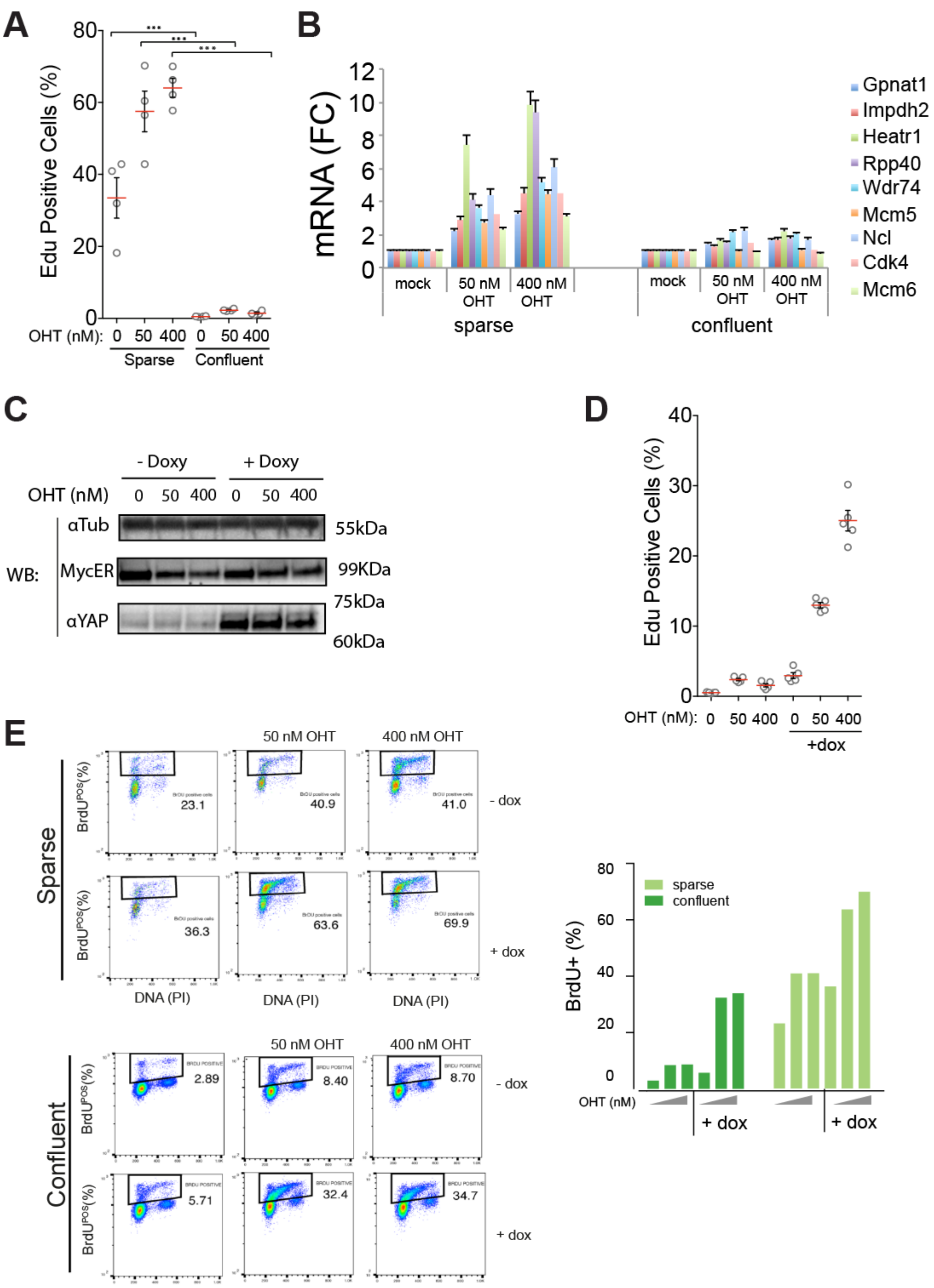

Supplemental Figure S1. Myc and YAP control cell cycle entry. Cell cycle entry of quiescent $3T9^{MycER}$ cells (A,B) and $3T9^{MycER;YAP}$ cells (C-E). (A,B) Sub-confluent (sparse) and confluent $3T9^{MycER}$ cells treated with the indicated doses of OHT to activate MycER. (A) Cell cycle entry assessed by EdU incorporation (relative to text figure 1A). (B) Expression of Myc target genes evaluated by RT-qPCR. (C-E) Cell cycle entry of quiescent $3T9^{MycER;YAP}$ cells. (C) Western blot analysis of confluent $3T9^{MycER;YAP}$ cells. Cell lysates were immunoblotted with antibodies against Myc, YAP or vinculin (vinc, used as a loading control). This analysis shows that activation of MycER does not lead to an increase in YAP levels. Similarly, induction of YAP does not lead to an increase of MycER expression, thus ruling out the possibility that an indirect regulation of the level of expression of the two TFs may account for their cooperative control of cell cycle entry. (D) Cell cycle entry of quiescent and confluent cells, assessed by EdU incorporation (relative to text figure 1A). (E) Cell cycle entry by FACS analysis of BrdU incorporation in either sparse or confluent cells. Right: Bar-graph of the percentage of $BrdU^{+}$ cells shown on the left.

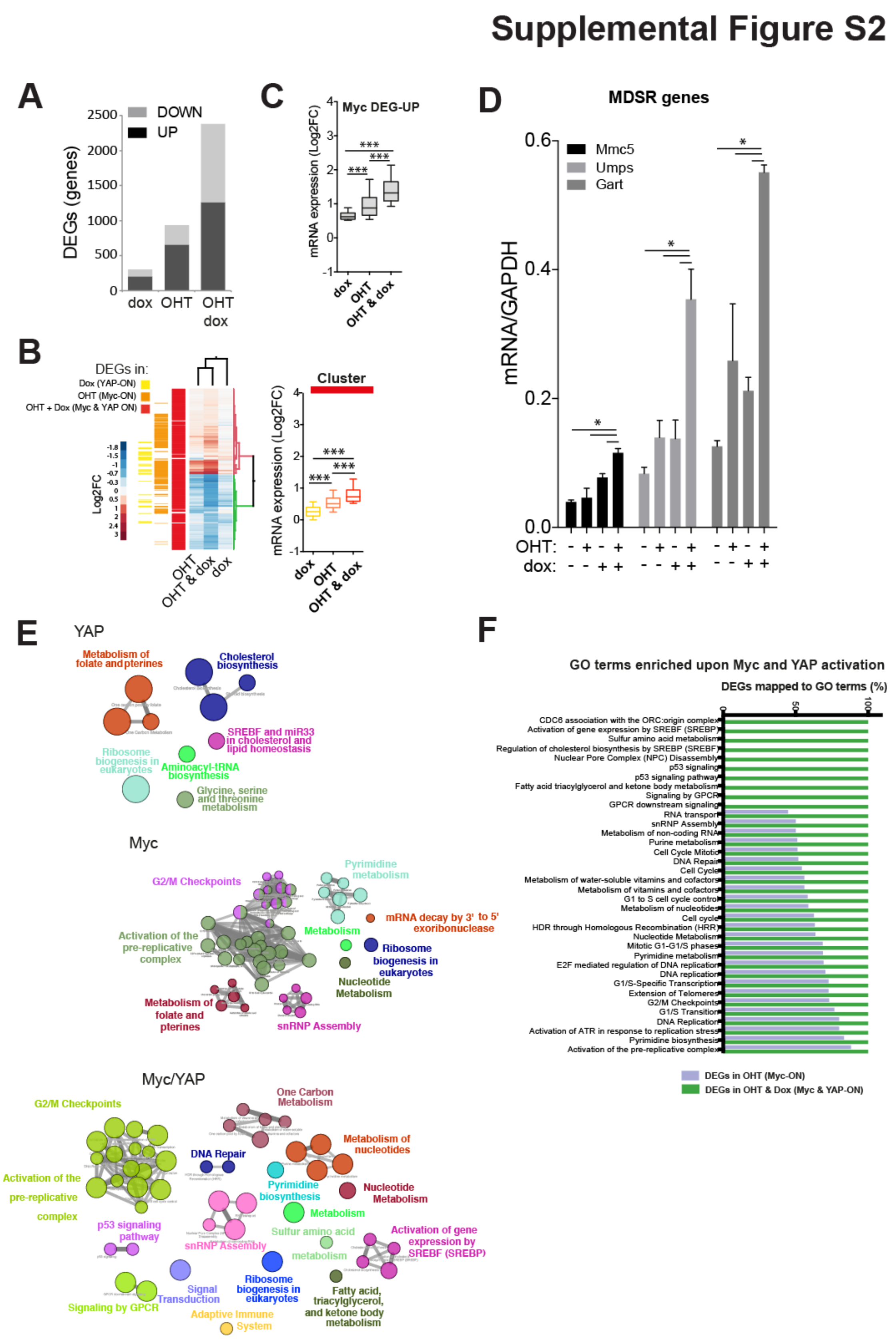

Supplemental Figure S2. Myc and YAP co-regulate genes linked to cell proliferation. RNA-seq analysis of 3T9$^{MycER;YAP}$ fibroblasts. (A) Cumulative histogram of the number of genes differentially expressed in the indicated conditions, compared to untreated cells. (B) Two-way

unsupervised hierarchical clustering based on the Log2FC of all the differentially expressed genes (DEGs) determined for all the described conditions, compared to controls (i.e. no activation of MycER or induction of YAP). DEGs in each condition are color coded on the side of the clustered heatmap. DEGs upon MycER activation (OHT) or MycER activation and YAP induction (dox) tend to cluster closer than the DEGs identified upon YAP induction alone (see column clustering). Nonetheless, the overall direction (but not the extent) of the transcriptional changes is rather consistent among the different conditions. Overall, this shows how YAP co-expression exacerbates a transcriptional program already elicited, albeit at low levels, when only MycER is activated. On the left, box plot showing the expression changes of genes of the red cluster (up regulated). (C) Box plot of the expression of the genes up-regulated upon MycER activation. This shows how Myc induced genes are further modulated by YAP over-expression. (D) RT-qPCR analysis of selected Myc dependent serum response genes (MDSR). The bar graph shows the mean of a technical triplicate. (E) Gene ontology map based on the DEGs determined upon either YAP induction (YAP), MycER activation (Myc) or both (Myc/YAP). (F) Bar graph showing the fractional enrichment of the GO terms associated to the DEGs determined upon Myc activation and YAP induction (Myc/YAP DEGs). While GO terms related to cell cycle are enriched in both Myc and Myc/YAP DEGs, upon Myc activation and YAP induction an higher fraction of genes is associated to these terms, thus showing a greater contribution to the transcriptional programs leading to cell cycle entry when both transcription factors are activated.

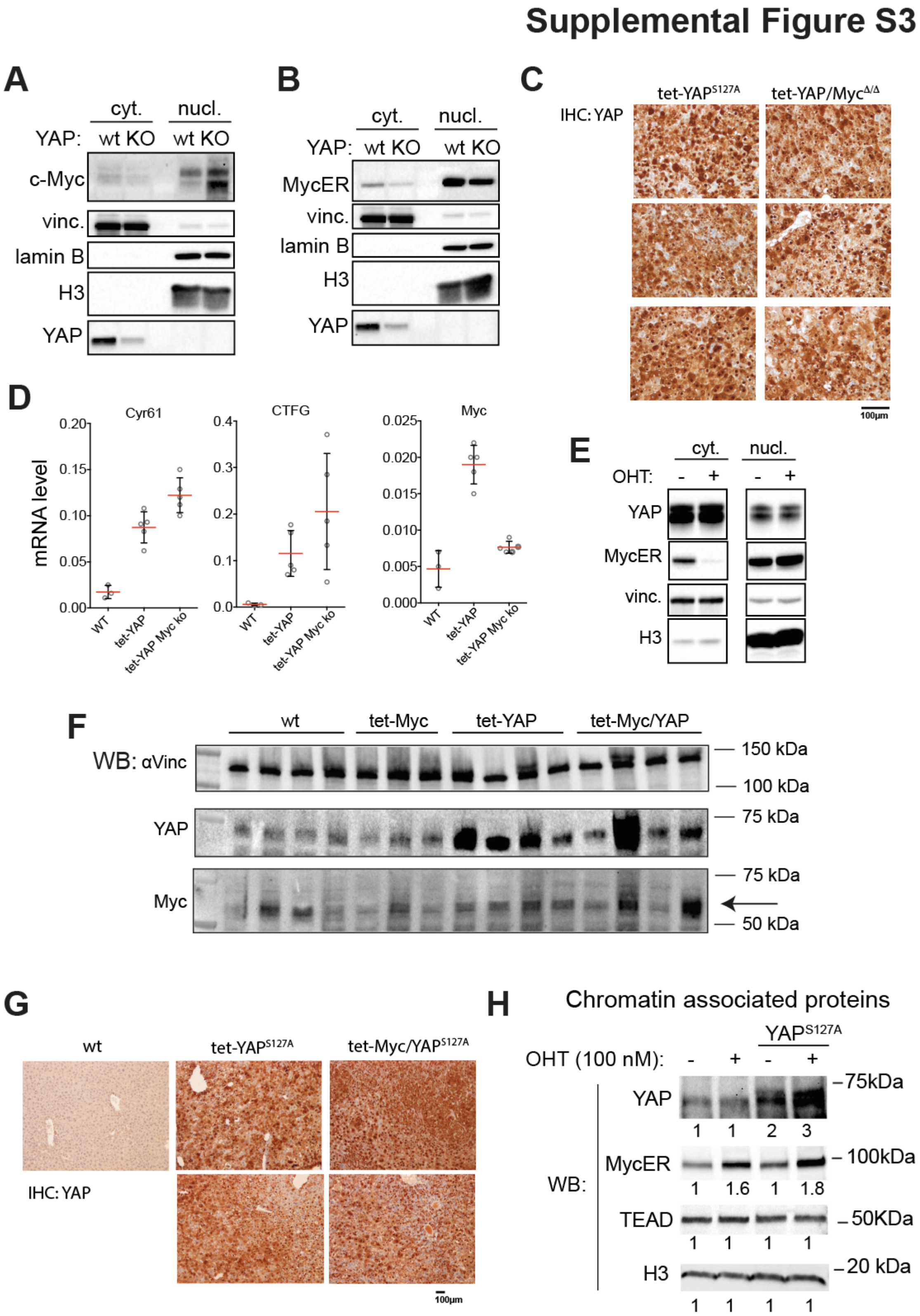

Supplemental Figure S3. Analysis of intracellular localization and chromatin association. (A,B) The intracellular localization of both endogenous Myc and MycER are independent from YAP expression. (A) Western Blotting analysis of the nucleo-cytoplasm partitioning of endogenous Myc in YAP$^{fl/fl}$ fibroblasts either wild-type (wt) or knock-out for YAP (ko).

Vinculin (vinc.) was used a marker for the cytoplasmic fraction, while lamin B and histone H3 (H3) as markers for the nuclear fraction. (B) MycER intracellular localization analyzed as in (A). (C) In vivo deletion of Myc (Alb-CRE mediated) does not affect YAP levels nor its intracellular localization. Immunohistochemical analysis of YAP localization in tet-YAP^{S127A} livers compared to tet-YAP^{S127A}; Myc$^{\Delta/\Delta}$ liver. (D) Myc deletion in the liver does not alter the expression of YAP targets genes (Ctgf and Cyr16) in adult tet-YAP livers. Graphs show normalized mRNA levels determined by RT-qPCR; each dot is an independent mouse. Red bar is the average, error bar is the stdv. (E) Activation of MycER in fibroblast does not affect intracellular localization of YAP^{S127A}. Western-blotting analysis as described in (A). (F) WB analysis of the levels of Myc and YAP in the liver of R26-rtTA tet-YAP, tet-Myc and tet-Myc;tet-YAP mice treated with doxycycline for 2 days. Due to its low expression level Myc is detected as a band of about 60kDa, marked by the arrow. Vinculin (vinc.) is used as an internal control for equal loading. (G) Induction of Myc in the liver does not affect YAP^{S127A} intracellular localization, as determined by IHC analysis. (H) Western blotting analysis of chromatin associated protein in 3T9MycER/YAP^{S127A} cells. Numbers indicate relative band intensity determined by densitometric analysis.

Supplemental Figure S4

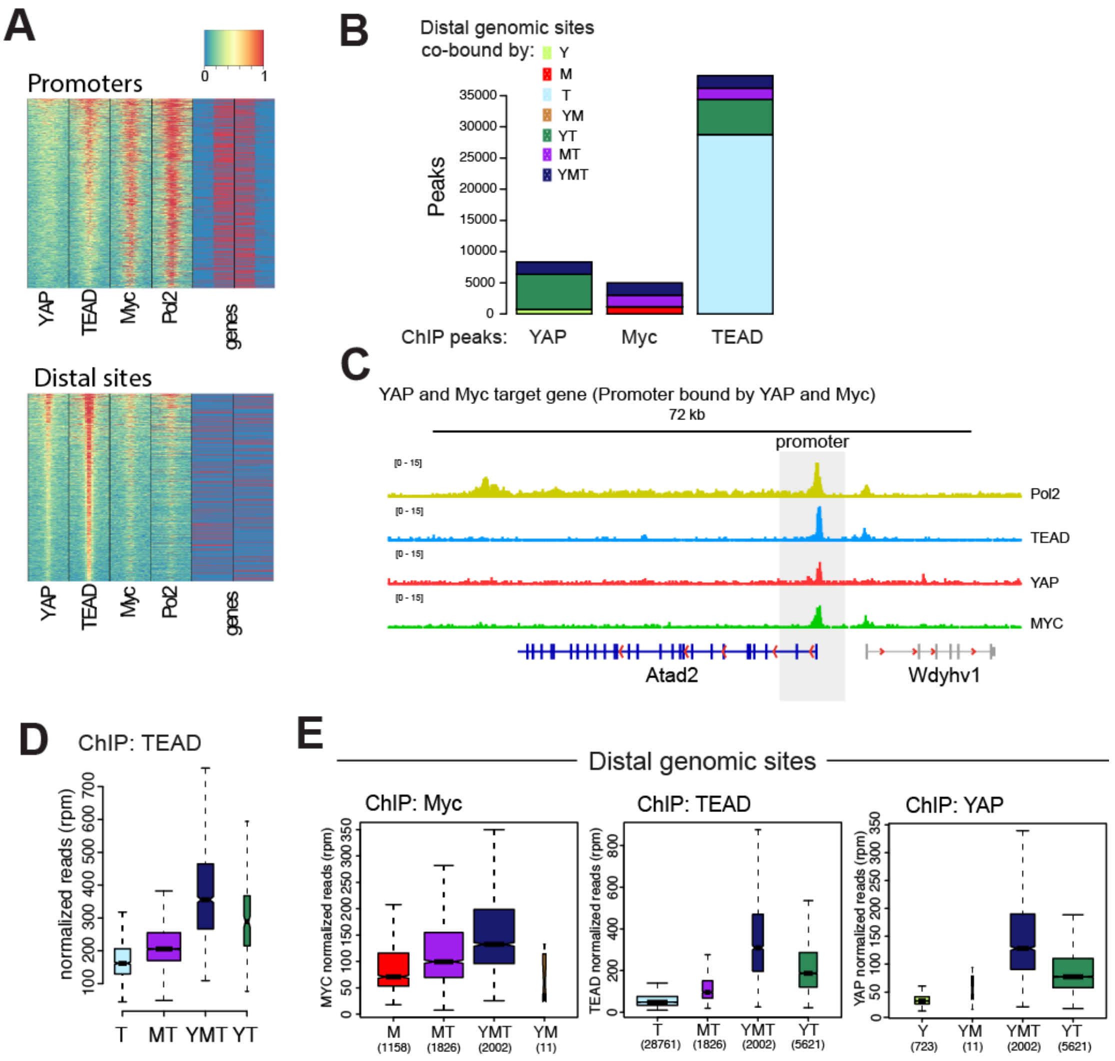

Supplemental Figure S4. ChIP-seq analyses of quiescent and confluent $3T9^{MycER;YAP}$ cells. Cells were kept in low serum and treated for 8 hours with OHT, to activate MycER, and doxycycline (dox) to trigger the expression of YAP^{S127A}. (A) Ranked heatmaps of the enrichments of YAP, TEAD, Myc and RNApol2 ChIP-seq peaks mapped to either promoters or distal sites. (B) Cumulative bar graph showing the number of distal sites bound by YAP (Y), TEAD (T) and Myc (M) in $3T9^{MycER;YAP}$ upon YAP induction and MycER activation. ChIP-seq peaks of each TF were subsetted and color coded based on the co-presence of the other transcription factors.

(C) Genome browser snapshot of Atad2, a gene co-bound at its promoter by YAP, Myc and TEAD. (D) Enrichment of promoter associated TEAD peaks. (E) Box plots of the enrichment of Myc (left panel), TEAD (center panel) and YAP (right panel) on distal peaks. Peaks are color coded as in (B). YMT peaks (i.e. peaks co-bound by YAP, Myc and TEAD) show the highest enrichment for all the TFs, compared to all the other subsets.

Supplemental Figure S5

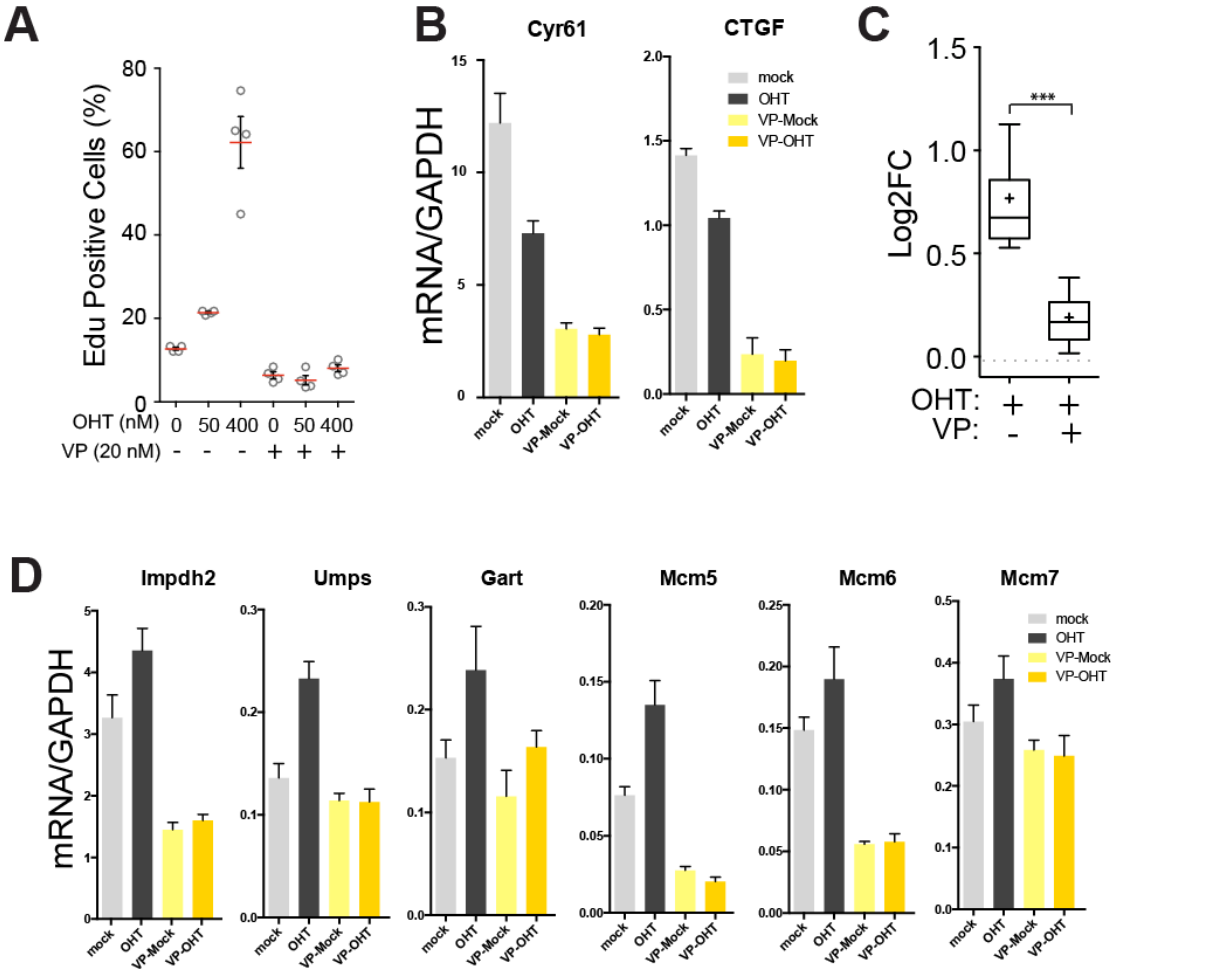

Supplemental Figure S5. The YAP inhibitor verteporfin inhibits Myc induced transcription and cell cycle entry. Serum starved sub-confluent 3T9 MycER cells were kept in low serum and treated with the indicated doses of OHT (to activate MycER) and the YAP inhibitor verteporfin (VP). (A) Myc induced cell cycle entry measured as EdU incorporation. Each circle represents the value of an independent biological replicate. Red bar is the average, error bar is the standard deviation. (B) RT-qPCR analysis of selected YAP target genes, showing the efficacy of VP. The bar graph shows the mean of technical triplicates and their standard deviation. (C) Box-plot of the expression (log2FC= log2 fold change) of MycER upregulated in the indicated conditions. (D) RT-qPCR analysis of selected MDSR genes. The bar graph shows the mean of technical triplicates and their standard deviation.

Supplemental Figure S6

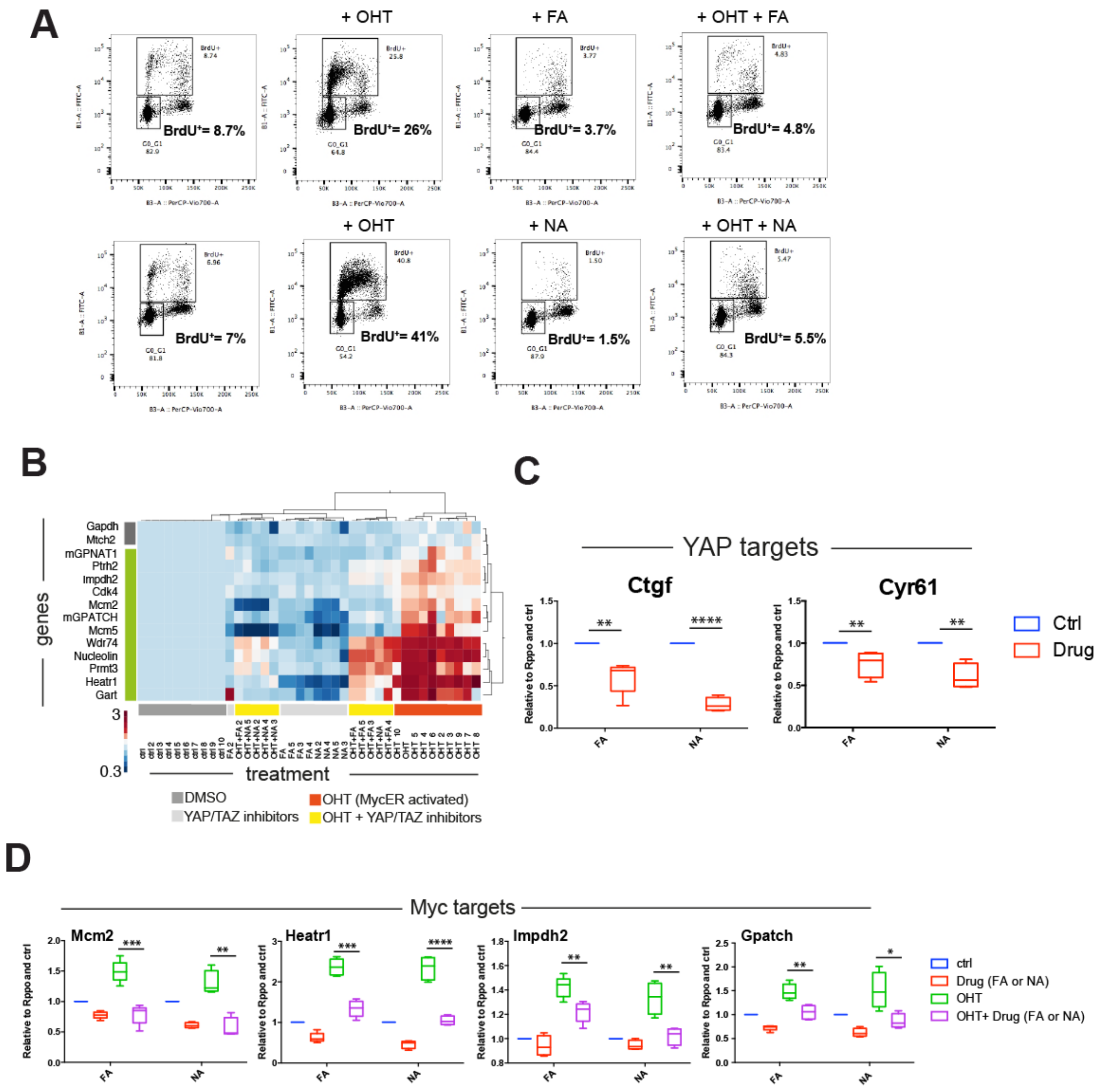

Supplemental Figure S6. YAP inhibition impairs Myc induced cell cycle entry and MDSR genes transcription. Serum starved sub-confluent 3T9^{MycER} cells were kept in low serum and treated with OHT (to activate MycER). YAP transcriptional activity was inhibited by either fuflenamic acid (FA) or niflumic acid (NA). (A) Cell cycle analysis by FACS, based on BrdU incorporation. (B) Unsupervised hierarchical clustering based on mRNA expression levels determined by RT-qPCR analysis. The heatmap reports the mean of a technical triplicate. Both fuflenamic and niflumic acid reduce the transcription of MDSR genes (green cluster, vertical). OHT samples (MycER activated) cluster apart from all the other conditions (orange cluster, horizontal). (C) RT-qPCR analysis showing the down-regulation of YAP/TAZ target genes upon either fuflenamic or niflumic acid treatment. (D) Box plot of the MDRS genes shown in (A). All box-plots show the result of at least four independent biological replicates.

Statistical analysis: multiple T-test, corrected for multiple comparisons (Holm-Sidak). * P value less than 0.01, ** P value less than 0.001,*** P value less than 0.0001.

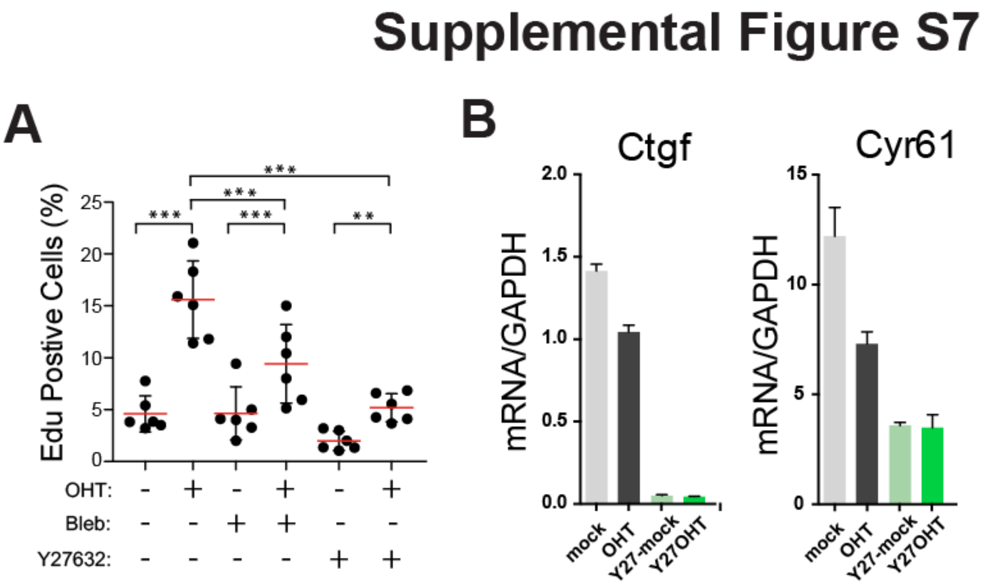

Supplemental Figure S7. Cytoskeleton inhibition impairs Myc induced cell cycle entry and transcription. Serum starved sub-confluent 3T9^{MycER} cells were kept in low serum and treated with OHT (to activate MycER) and either the ROCK inhibitor Y276632 (Y27) or blebbistatin (bleb). (A) Graph of the percentage of EdU positive cells determined for each biological replicate (n=6). Red bar is the average, error bar is the standard deviation. *** P value less than 0.001. (B) RT-qPCR analysis showing the down-regulation of YAP/TAZ target genes.

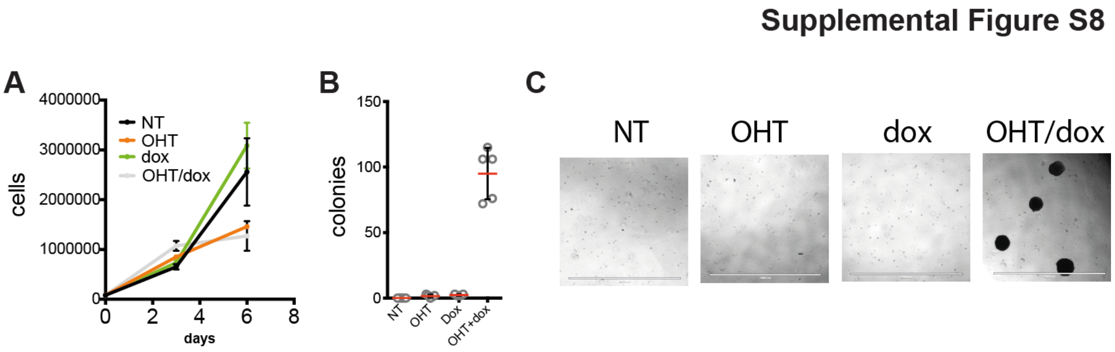

Supplemental Figure S8. The fetal liver derived BMEL cell line was infected with MycER and pSLIK-YAP^{S127A}. Cells were grown in the indicated conditions in the presence of OHT, to activate MycER, and doxycycline (dox) to trigger the expression of YAP^{S127A} (A) Growth curves of BMEL grown in adherent conditions (n=3, error bar is the stdv). (B-C) Anchorage independent growth assay. (B) dot-plot of the number of colonies detected after 14 days of culture (n=5; red bar is the average, error bar is the stdv). (C) Representative pictures of cell colonies scored in (B).

Supplemental Figure S9

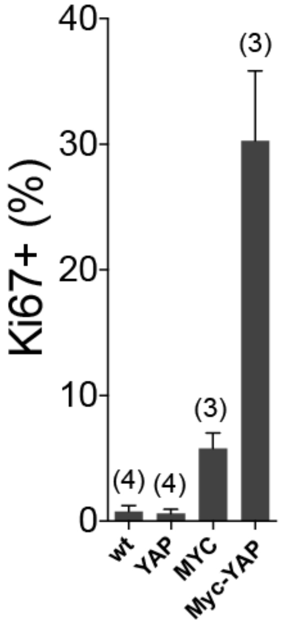

Supplemental Figure S9. Myc and YAP induce proliferation in the adult murine liver. R26-rtTA tet-YAP, tet-Myc and tet-Myc;tet-YAP mice were treated with doxycycline for 2 days to induced the transcription of Myc, YAP or both. Bar graph showing the proliferation levels in the liver, as assessed by the percentage of nuclei showing a positive signal for Ki67 determined in immuno-histochemical analysis. The number of mice analyzed for each condition is indicated in brackets. Data are reported as average and the error bar is the standard deviation.

Supplemental Figure S10

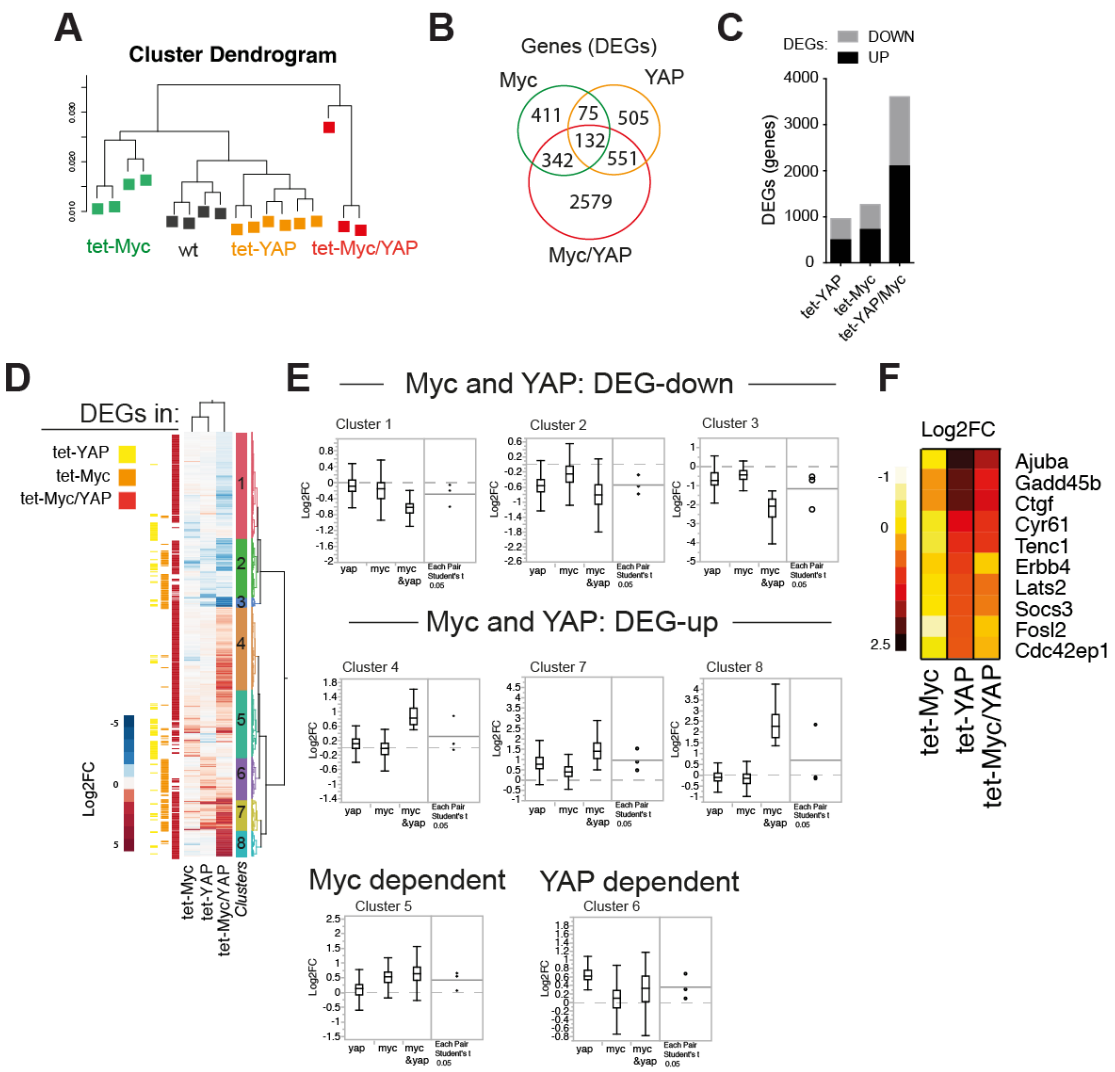

Supplemental Figure S10. RNA-seq analysis upon short-term induction of YAP and Myc in the liver. R26-rtTA tet-YAP, tet-Myc and tet-Myc/YAP mice were feed with doxycycline for 2 days to induced the transcription of Myc, YAP or both. (A) Unsupervised clustering based on the RNA-seq analysis (RPKM). tet-Myc/YAP livers cluster apart from all the other conditions analyzed. (B) Venn diagram analysis of the DEGs identified in the indicated genotypes. Numbers are the genes belonging to the different categories. (C) Bar graph of DEGs identified by RNA-seq. (D) Hierarchical clustering (two-way) based on the differential expression of the DEGs reported in (C). DEGs are indicated (color coded) on the side of the heatmap. (E) Box-plots and statistical analysis of the cluster of genes identified in (B). Clustering identifies genes that are up-regulated (clusters 4,7 and 8) or down-regulated (clusters 1,2 and 3) by both YAP and Myc as well as genes that are up-regulated by Myc (cluster 5). Genes up-regulated by YAP (cluster 6) are down-modulated by Myc co-expression. (F) Heatmap of the expression of selected YAP target genes.

Supplemental Figure S11

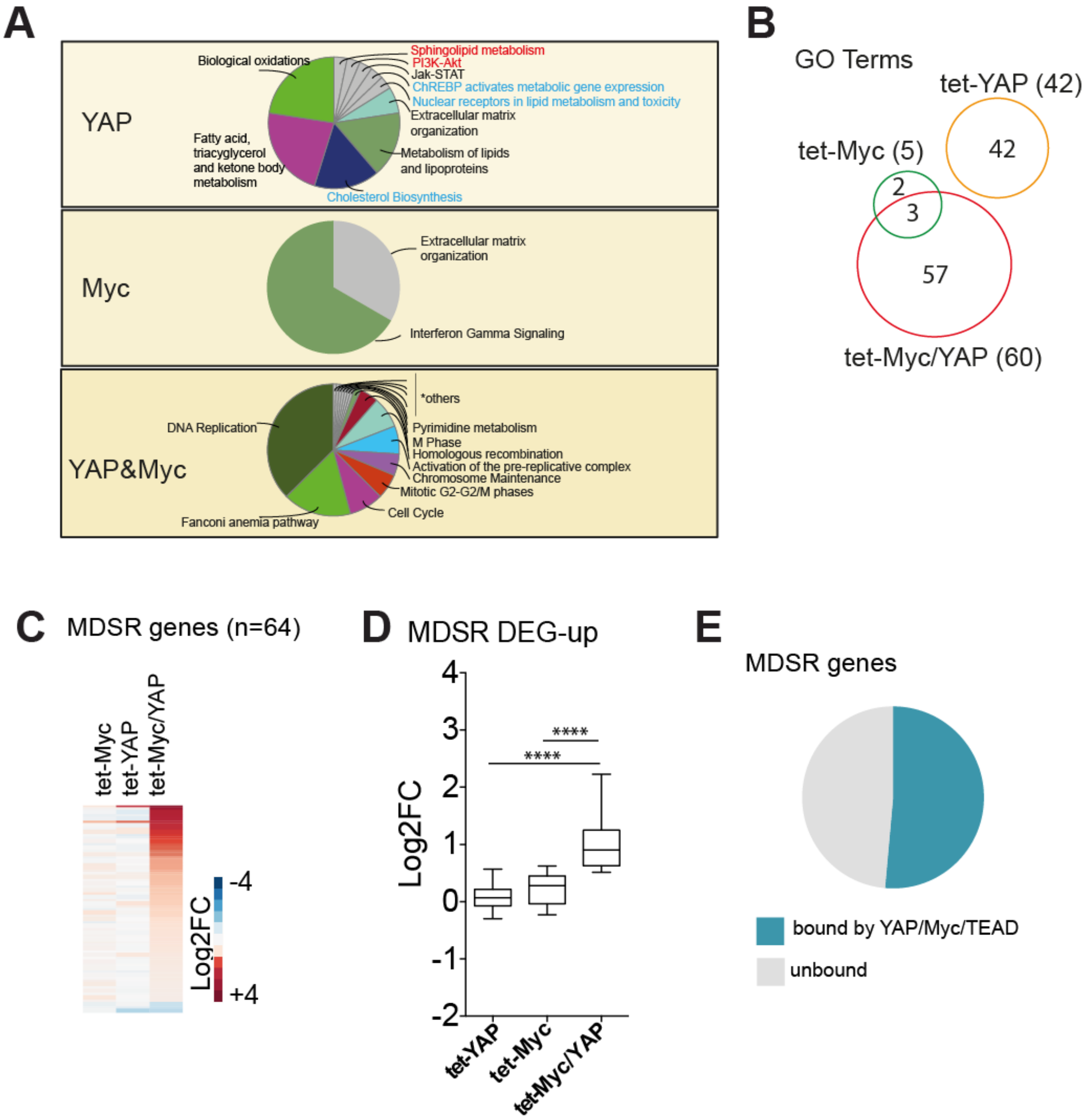

Supplemental Figure S11. Myc and YAP regulate the expression of liver genes linked to cellular proliferation. (A) Gene Ontology analysis of the DEGs identified by RNA-seq upon induction of YAP, Myc or both. (B) Venn diagram based on the GO analysis shown in (A). (C) Ranked heatmap of the relative expression (Log2FC) of Myc dependent serum response genes (MDSR) that are DEGs in tet-Myc/YAP liver upon short-term induction of both TFs. (D) Box plot of the expression level of the up-regulated MDSR genes upon induction of Myc, YAP or both. (E) Pie chart showing the fraction of MDSR genes co-bound at their promoters by Myc,YAP and TEAD in tet-Myc/YAP mice.

Supplemental Figure S12

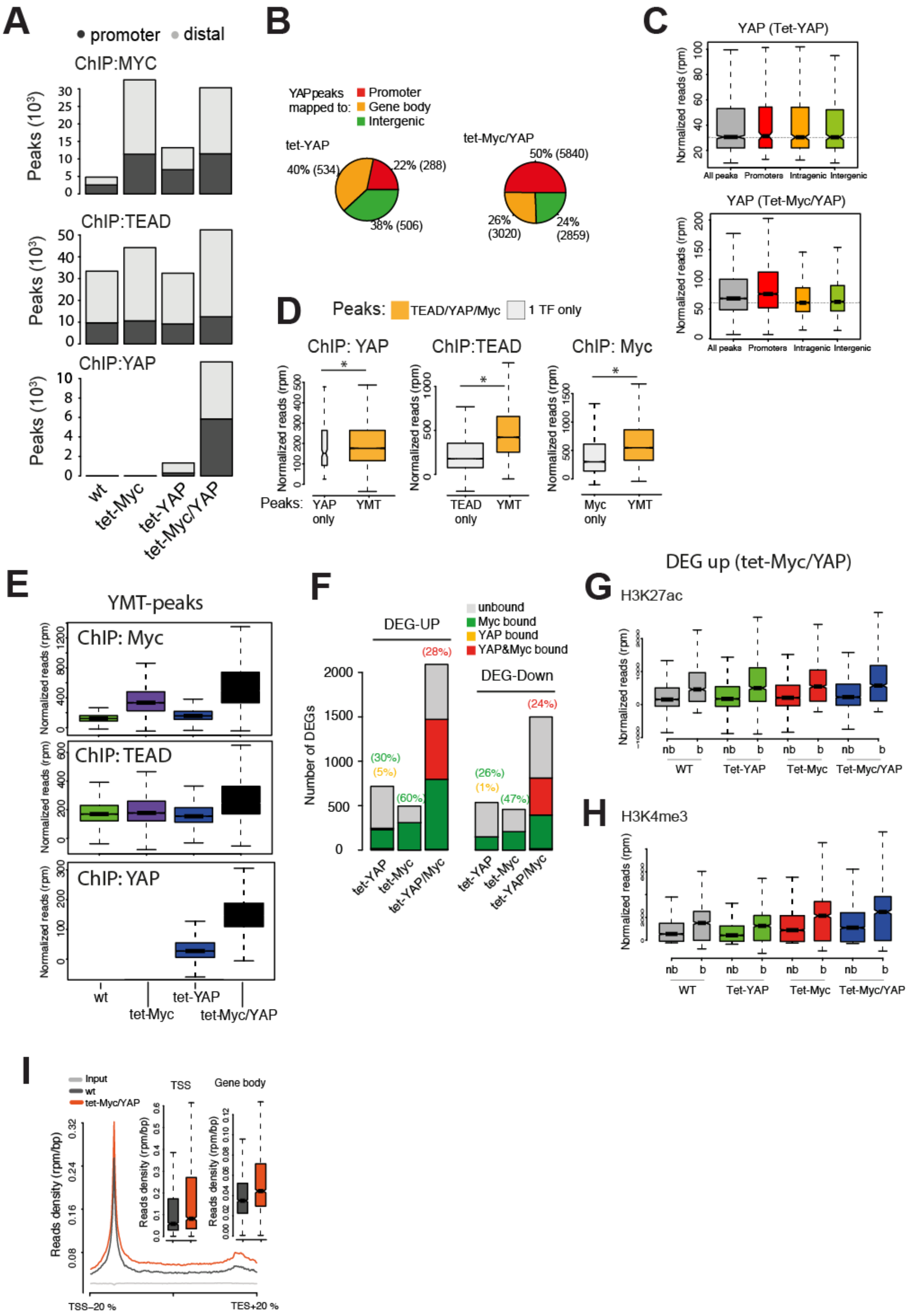

Supplemental Figure S12. Summary of the ChIP-seq analyses upon short-term induction of YAP and Myc in liver. (A) Number of ChIP-seq peaks determined for the indicated transcription factor in the different genetic backgrounds. Peaks are annotated as distal (dark

gray) or promoter associated (light gray). (B) Pie chart showing the genomic distribution of YAP ChIP-seq peaks. (C) Box plots showing the enrichment of YAP peaks identified in tet-YAP (left panel) and tet-Myc/YAP mice (right panel). Peaks were subsetted based on their genomic location, as described in (B). Promoter associated YAP peaks (in red) identified in tet-Myc/YAP mice have a higher enrichment compared to the distal YAP peaks (i.e. intragenic and intergenic peaks) mirroring the higher enrichment of Myc at promoters. (D) Box plot of the enrichment of YAP, Myc and TEAD in peaks where the three TFs are present (YMT) compared to the peaks where only one TF is detected. (E) Box-plot of the enrichment of each TF in the YMT regions, determined in the different genetic backgrounds. (F) Bar graph of the binding of either YAP, Myc or both to the TSS of DEGs subsetted as up-regulated (DEG-up) or down-regulated (DEG-down). The percentage of bound DEGs is reported within brackets. (G,H) Box plot of H3K27Ac (I) and H3K4me3 (H) enrichments measured at promoters of up-regulated genes (DEG-UP) identified in tet-Myc/YAP mice. Each box-plot shows the enrichment for promoter bound (b) or not-bound (nb) by both YAP and Myc in tet-Myc/YAP mice. DEG-UP genes have higher level of H3K4me3 and H3K27Ac at their promoters when bound by both YAP and Myc. (I) RNApol2 distribution along DEG-up genes bound by both YAP and Myc. RNApol2 enrichment at TSS and gene body are reported in the inset. (I) RNApol2 distribution along DEG-up genes bound by both YAP and Myc. RNApol2 enrichment at TSS and gene body are reported in the inset.

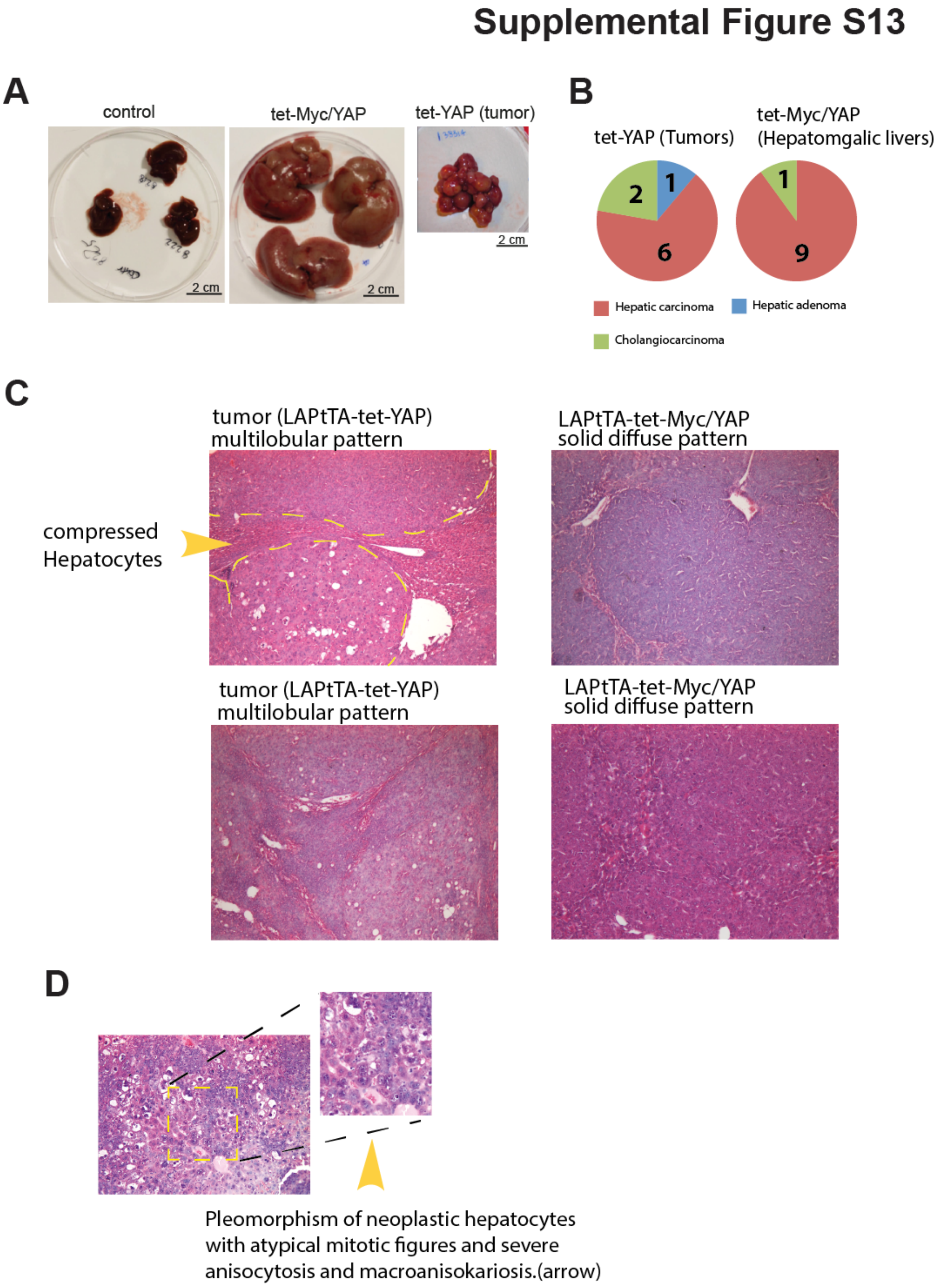

Supplemental Figure S13. Long-term induction of YAP and Myc in the liver.

Mice of the indicated genotypes were kept on a doxycycline free diet (to induce the expression of tet-Myc and tet-YAP transgenes in the liver) since weaning. (A) Representative liver images of livers of the indicated genotype. (B) Pie chart of the classification of liver sections from tet-Myc/YAP (hepatomegalic livers) and tet-YAP (nodular livers) sampled at the endpoint of the experiment shown in figure 4B. tet-YAP sections were classified mainly

as Hepatocellular carcinomas with few Adenoma and Cholangiocellular carcinomas. Liver sections from tet-Myc/YAP mice were all classified as hepatocellular carcinomas, with a solid pattern of growth. (C-D) Haematoxylin Eosin stained sections of representative livers of the indicated genotypes. Coherent with the presence of macroscopic liver noduli, tet-YAP sections showed multi-focal irregular nodular proliferation of atypical cells (B), while tet-Myc/YAP livers showed a solid pattern of growth with neoplastic lobules divided by a minimum amount of fibrovascular connectival stroma (B). Cells displayed severe macroanisocytosis and macroanisokariosis, with a lobular proliferation of atypical hepatocytes starting from central veins (C).

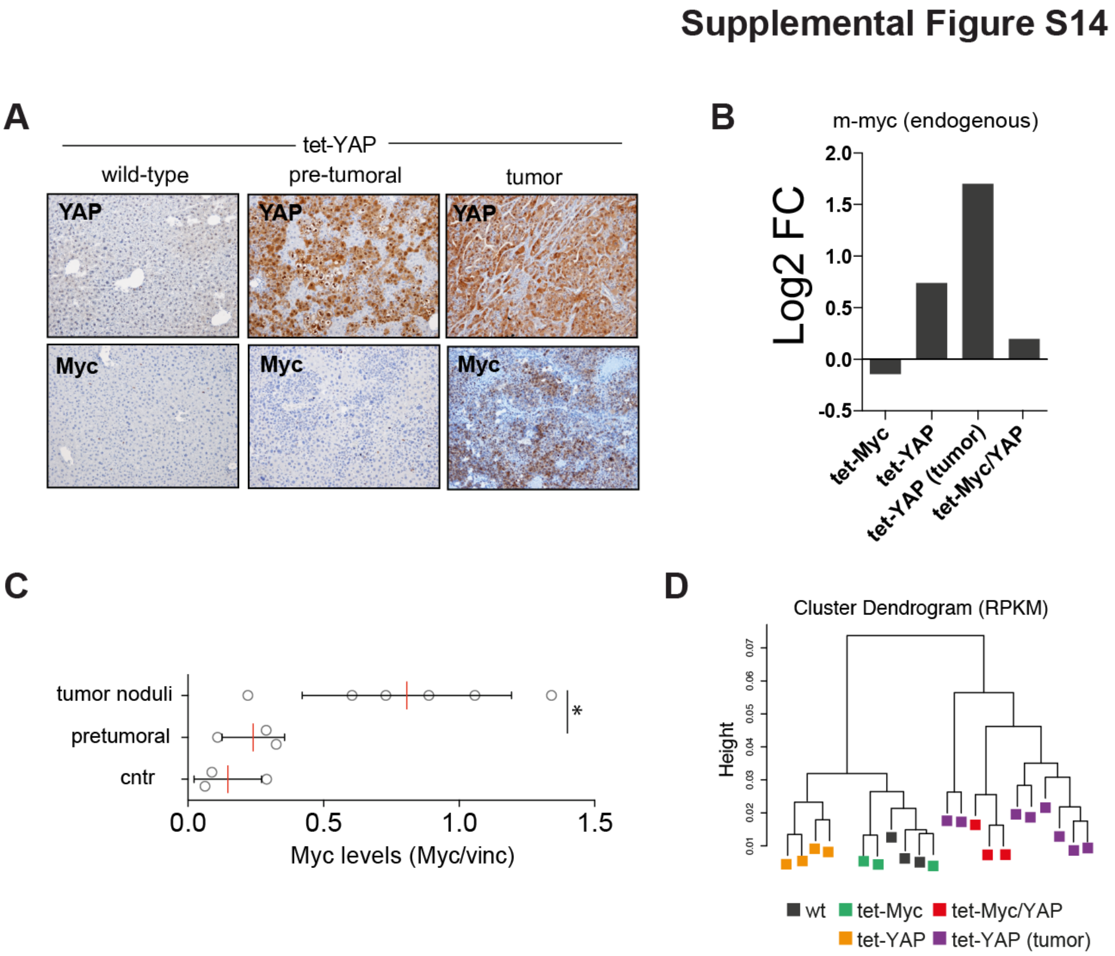

Supplemental Figure S14. (A) IHC analysis of Myc and YAP levels in the liver, during YAP driven tumorigenesis. HCC-like lesions show higher levels of Myc compared to dysplastic lesions observed at the pre-tumoral stage in tet-YAP mice. (B) Myc mRNA levels in liver of tet-Myc, tet-YAP (pretumoral), tet-YAP induced tumors (T) or in tet-YAP/Myc mice. In line with Myc ability to repress its own expression, induction of tet-Myc results in low transcription of the (endogenous) c-Myc locus observed in tet-Myc and tet-Myc/YAP hepatocytes. (C) Dot-plot shows Myc protein levels as determined by densitometric analysis of the western blot shown in text figure 4C. Data are normalized to vinculin. * P value = 0.01. (D) Clustering based on normalized mRNA expression (RPKM).

Supplemental Figure S15

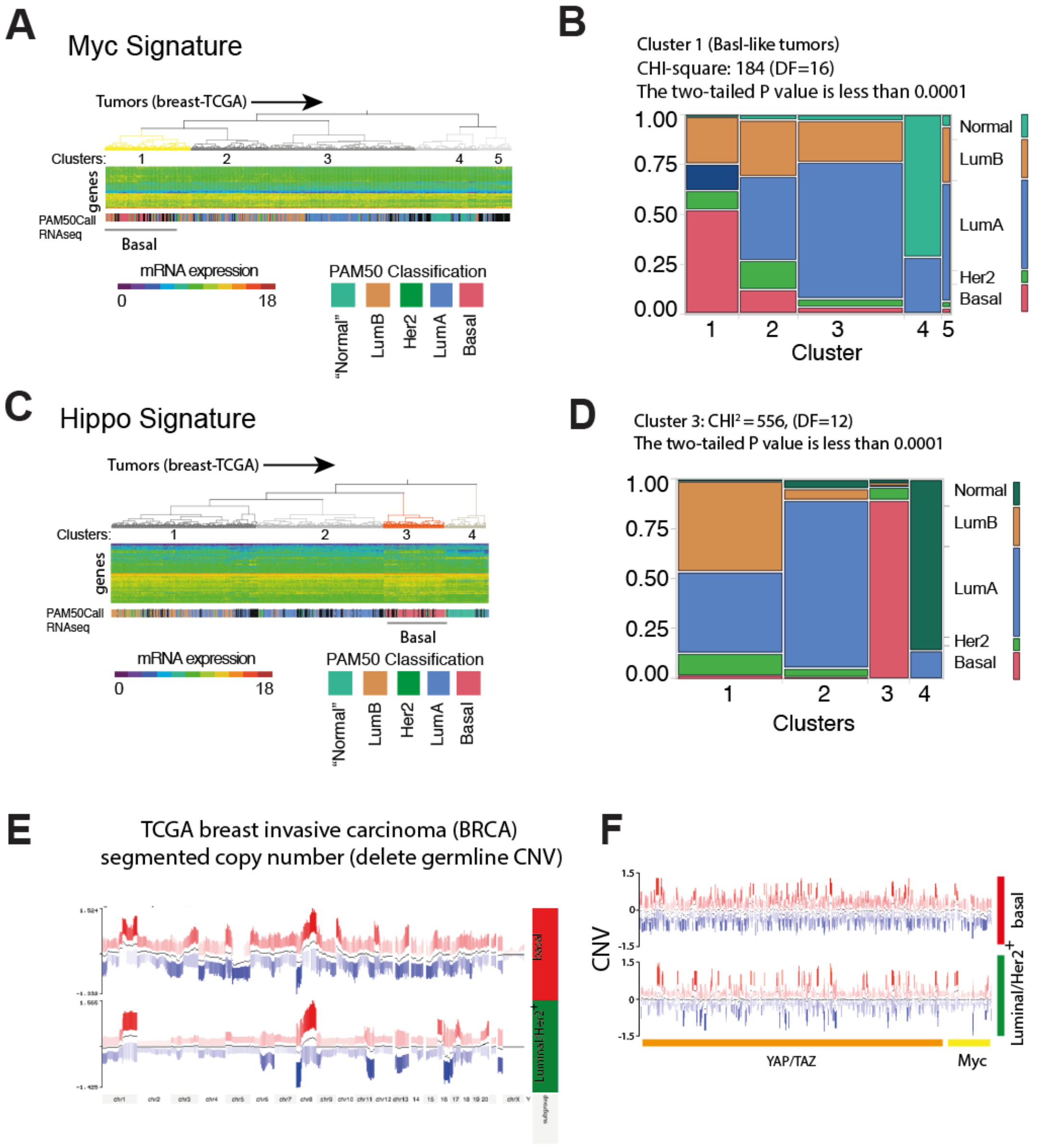

Supplemental Figure S15. Basal-like aggressive carcinomas can be predicted based on the expression of a Myc signature (Ji et al., PloS One (2011)).

(A,B) Myc signature can stratify basal-like breast tumors apart from all the other classes. (B) Two-way clustering analysis based on normalized RNA expression (data taken from TCGA_BRCA dataset), line blow reports the classification of the different tumors, based on the PAM_50 RNA-seq call (color coded as indicated). As indicated in yellow, the majority of basal like cancer cluster apart from all the other breast tumors. (B) The contingency analysis (mosaic plot) confirms the predictive power of the Myc signature. (C,D) As a way of comparison the same analysis was performed using the YAP/TAZ signature from Zanconato et al., Nat cell. Biol, (2015). As reported, the YAP/TAZ signature stratifies basal-like breast

cancers. (E) Genome wide copy number variation analysis (CNV) of basal-like breast tumors compared to Luminal/Her2+ breast tumors. Copy number gains are reported in red, losses in blue. This shows that both Basal-like and Luminal/Her2+ breast tumors are equally enriched for tumors with chromosome 8 amplification (i.e. this is where Myc is located), thus overexpression of Myc targets in basal-like breast tumors can not be explained by a higher frequency of Myc amplification. (F) CNV of the genomic loci encoding the genes present in either the Myc or the YAP/TAZ signatures. This shows that their differential expression in basal-like tumors is not linked to CNV of such genes in this tumor type.

Supplemental Figure S16

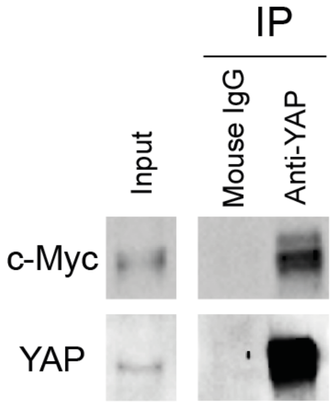

Supplemental Figure S16. Myc co-immunoprecipitates with YAP. Cell lysates (input) prepared from MDA-MB-231 cells were immuno-precipitated with anti-YAP or control IgG and analyzed by western blotting using the indicated antibodies.

METHODS

Mice strains. Tet-YAP mice (Col1A1-YAP^{S127A} transgenic mice) were kindly provided by Jonas Larsson[28]. tet-MYC transgenic mice were a kind gift from Martin Eilers(van Riggelen et al. 2010). For liver-specific transgene expression, mice were crossed with LAP-tTA mice expressing the tTA tetracycline transactivator under the control of the LAP promoter (B6.Cg-Tg(tTALap)5Bjd/J; purchased from Jackson Laboratories). To suppress transgene expression, LAP-tTA transgenic mice were kept under continuous administration of food supplemented with doxycycline (625mg/Kg). For long-term activation, 8 weeks old mice were shifted to a regular diet (i.e. doxycycline free). For short-term transgene activation, mice were crossed with animals expressing the rtTA tetracycline transactivator under the control of the Rosa26 ubiquitous promoter[28]. To activate transgene expression, mice were feed with doxycycline-supplemented food for 2 days. All experiments were performed in accordance with the Italian law (D.L.vo 116/92 and following additions). Flox-Myc mice (C57/Bl6) were a kind gift of Andreas Trumpp; Alb-CRE mice were purchased from the Jackson lab (B6.Cg-Tg(Alb-cre)21Mgn/J, stock #003574); Yap$^{fl/fl}$ mice were purchased from the Komp consortium (Yap1$^{tm1a(KOMP)Mbp}$).

Cell culture. 3T9^{MycER} murine fibroblast (Sabo et al. 2014) were infected with pSlik-YAP^{S127A} retroviruses and selected with 100ug/mL Hygromycin, for one week. For subsequent experiments, cells were grown in DMEM medium supplemented with 10% tetracyclin-free serum, penicillin/streptomycin and 2mM L-Gln. MycER was activated by the addition of 4-hydroxytamoxifen (OHT, 20-400nM), while YAP^{S127A} was induced by 2 µg/ml doxycycline. Ethanol was used as vehicle and mock treatments. For experiments in hyperconfluent cultures, cells were initially seeded at a defined density (5000 cells/cm^2) and grown for 7 days (confluency was typically reached within 2-3 days). When indicated, cells were starved for 48h in low serum (DMEM medium supplemented with 0.1% TET-free serum, penicillin/streptomycin and 2mM L-Gln). BMEL cell line (murine bipotent embryonic liver cells, Strick-Marchand et al, 2002) were infected with pSlik-YAP^{S127A} and pBABE-MycER retroviruses (Sabo et al. 2014). Cells were grown in RPMI supplemented with 10% FBS, IGF II (30ng/ ml, Peprotech), EGF (50ng/ml, Peprotech), insulin (10µg/ml, Peprotech) on collagen coated plates (collagen type I Rat Tail 1mg/ml, Invitrogen/BD). Anchorage independent growth was carried out in the same media, mixed 1:1 with Methycellulose (MethoCult™, SF M3236, STEMCELL technologies). All cell lines were tested and resulted negative for Mycoplasma.

Immunoistochemical analysis. Fresh liver and dissected noduli were fixed with 4% formaldehyde in PBS, (overnight at 4°C), paraffin embedded and sectioned at 6-8 μM of thickness. For histology, sections were counter-stained with Haematoxylin and eosin. Immunohistochemistry was performed as previously described (Campaner et al. 2010).

Cell cycle analysis by fluorescence microscopy. Cells were grown on sterile coverslips (5000 cells/cm^2) until the desired confluency was reached and then serum-starved for 24-48 hours. Then, cells were pulsed with EdU (final concentration 10uM) and stimulated with OHT and/or Doxycycline for 16 hours. EdU staining was performed according to the manufacturer's instructions (Click-iT Plus Edu Alexa Fluor 647 Imaging Kits, Thermofisher). Evaluation of cells in S-phase for the different treatments was performed blindly. For each condition, at least 6 random fields, with a total of 100-200cells/field, were acquired. Image analysis was performed with the ImageJ software (Schneider et al. 2012).

Cell cycle analysis by FACS. Cells were either labelled in continuous for 16 hours or pulsed labelled with BrdU (33uM) one hour before collection. After harvesting, cells were fixed in ethanol O/N at 4°C. DNA denaturation was performed with 2N HCl solution and the reaction was blocked with 0.1M Sodium Borate (Na2B4O7 pH 8.5). The cells were then collected and stained with anti-BrdU mouse antibody (BD-Biosciences), for 1h at room temperature and then with an anti-mouse IgG-FITC conjugated antibody. After one washing step, cells were incubated O/N at 4°C in PBS supplemented with Propidium Iodide (2.5 µg/mL) and RNaseA. The acquisitions were performed with a FACS Calibur. The analysis was performed with FlowJo software.

Chromatin-binding assay. Confluent cells were starved with 0.1% serum for 48h and then treated with OHT 100nM for 8h. Cells were then fixed with 1% formaldehyde, collected in 0.5% SDS buffer and sonicate. Chromatins were resolved on acrylamide gel and blotted with anti-Myc and Yap antibodies.

Chromatin immunoprecipitation. For Myc, TEAD, Histone Marks and RNAPol2 ChIP, 3T9 fibroblasts or dissected liver/tumors were fixed with 1% formaldehyde in PBS and quenched with 0.125M of Glycine. For YAP ChIP, fixation was performed by a double step approach with 0.5 M DSG (Di-(N-succinimidyl)-glutarate) for 45 min and then 1% formaldehyde (FA) in PBS 12 min. Fixed cells or tissues were further processed as previously described (Sabo et al. 2014). For ChIPq-PCR, 6 µL of DNA diluted 1:6 was used to perform real-time PCR using FAST SYBR Green Master Mix (Applied Biosystems). For sequencing purpose, 2-5 ng of ChIPed DNA was prepared for HiSeq2000 sequencing with TruSeq ChIP sample preparation kit (Illumina) following the manufacturer's instruction.

Immunoblot analysis. Cells or liver tissue/tumoral noduli were lysed with RIPA buffer (20 mM HEPES pH7.5, 300 mM NaCl, 5 mM EDTA, 10% Glycerol, 1% Triton X-100) supplemented with MINI-complete Protease Inhibitor Cocktail Tablets (Roche) and phosphatase inhibition (0.4 mM ortovanadate, 10 mM NaF) and sonicated (Branson). Cleared lysates were quantified by Bradford assay, run on a SDS-page and immunoblotted with the indicated primary antibodies. Chemiluminescent detection after incubation of the membranes with the appropriate secondary antibody, was done through a CCD camera using the ChemiDoc System (Bio-Rad).

Co-Immunoprecipitation. MDA-MB-231 cells were lysed by briefly sonication in buffer A (50 mM Tris-HCl pH 7.5, 150 mM NaCl, 1 mM EDTA, 0.5% NP40, 10% glycerol supplemented with protease and phosphatase inhibitors). Cleared cell extracts were immunoprecipitated with 2 µg of the indicated antibody.

Nucleus/Cytoplasm fractionation. Cells were resuspended in 200 ul of RSBS (10mM Tris/HCl pH 7.5, 10mM NaCl, 3 mM MgCl2) and incubated at 4°C for 5 minutes. After addition of NP40 (final concentration of 0.2%) cells were centrifuged for 10 minutes at 1200 g. The supernatant was the cytosolic fraction. The pellet was washed twice with 20 volumes of washing buffer (0,88 mM Sucrose, 5 mM MgAcetate, supplemented with Protease/Phospatase inhibitors). Pellet (nuclear fraction) was resuspended in Laemli sample buffer and sonicated. Fractions were analysed by western blotting (normalized by cell equivalents).

Antibodies. The following Antibodies were used for ChIP: H3K4me1 (Abcam, ab8895), H3K27ac (Abcam, ab4729), H3K4me3 (Active Motif, #39159), MycN262 (Santa Cruz, sc 764) and RNAPol2 N20-X (Santa Cruz, sc-899), YAP 63.7 (Santa Cruz, sc-101199), Yap H125-X (Santa Cruz, sc-15407), TEAD (*ARP38276_P050, Aviva Systems Biology*). Normal rabbit IgG (Santa Cruz, sc-2027) was used as background control. For western blot: Myc Y69 (Abcam, ab32072), Vinculin (Sigma, V9264), YAP (Cell Signalling #4912)

Chemicals. Y27632 (used 50µM), Blebblistatin (used 25µM) Verteporfin (used 20µM), Niflumic acid (used 150 µM) and Flufenamic acid (used 150 µM) were purchased from SIGMA.

Plasmids. The full-length cDNA of YAP^{S127A} PCR amplified from pQCXIH-Flag-YAP^{S127A} plasmid (addgene #33092) was cloned into pSLIK hygro plasmid using the GATEWAY system (Life Technology, USA).

Computational analysis NGS data analysis. ChIP-Seq and RNA-Seq experiments were analyzed using HTS-flow (Bianchi et al. 2016), a novel framework for NGS data analysis.

ChIP-seq and RNA-Seq reads sequenced with the Illumina HiSeq2000 were filtered using the fastq_masker (setting the options to -Q33 -q 20 -r -N -v -i) and duplicate reads were excluded from the analyses. This tool is part of the FASTX-Toolkit suite (http://hannonlab.cshl.edu/fastx_toolkit/). Their quality was evaluated and confirmed using the FastQC application. (http://www.bioinformatics.babraham.ac.uk/projects/fastqc/).

Analysis of ChIP-Seq data. NGS reads were aligned using with the BWA v.0.6.2 tool(Li and Durbin 2009). Alignment was performed with BWA-MEM and with default settings, using mm9 genome assembly. Peaks were called with the MACS v.2.0.9 software(Zhang et al. 2008). Peaks' p-value threshold was set to 10^{-8} for all the samples. Negative peaks were found by MACS on the input samples, using the ChIP as reference. Normalized reads count within a genomic region was determined as the number of reads per million of library aligned reads (rpm) that were subtracted by the input normalized reads, using 'compEpiTools', a bioconductor R package(Kishore et al. 2015). Peak read density (reads per million of reads per base pair) for a particular region was determined as the ratio between the normalized reads count and the length of the region expressed in base pair.

Analysis of RNA-Seq data. RNA-seq paired end reads were aligned using tophat v. 2.0.8 software, with the following options: -r 170 -p 8 --no-novel-juncs --no-novel-indels --library-type fr-unstranded. Differential gene expression analysis was carried out using DESeq2 R bioconductor package(Love et al. 2014). Up-regulated genes (DEG-UP) were defined by an adjusted p-values of less than 0.05 and a Log2 fold change > 0.5. Down-regulated genes (DEG-down) were defined by an adjusted p-value less than 0.05 and a Log2 fold change < -0.5. The rest of the genes were defined as not deregulated (no DEG).

Definition of promoter, intragenic and intergenic regions. In order to assess if a specific ChIP-seq peak mapped to a promoter, a gene body or to an intergenic region, the following criteria were applied: regions that overlap with at least one bp with any promoter (defined as genomic region [-2000; +1000] bp spanning TSSs, transcription start sites), were considered as belonging to promoters; regions that weren't promoters but had at least 1 bp overlapping with any gene body were considered intragenic. The remaining regions (that did not overlap either with promoters or gene bodies) were considered intergenic. Annotations were performed with the R annotation package TxDb.Mmusculus.UCSC.mm9.knownGene of Bioconductor. For the analysis of RNAPol2 distribution along genes, the ± 300 bp interval around annotated transcription start sites was considered as TSSs, and the region between TSS+301 bp and the end of the gene as gene body. RNAPol2 signal profiles were plotted expanding the genes by 20% of their length upstream and downstream and then divided into

150 bins, for which the average input-subtracted reads were counted. Reads were normalized both for library size and gene length, using "GRcoverageInbins" function of compEpiTools R package(Kishore et al. 2015).

Other bioinformatic and statistical analysis. Gene ontology analysis was performed using the ClueGO plug-in (Bindea et al. 2009) for cytoscape (Shannon et al. 2003). Gene set enrichment analysis (GSEA) was performed using the C2 gene sets from the MSigDB, either online (http://software.broadinstitute.org/) or with the GSEA desktop software (Subramanian et al. 2005). Clustering analysis and heatmaps of RNAseq expression data were performed with R statistical software and the JMP software (SAS Institute Inc.). The analyses on breast tumors were performed on TCGA invasive breast carcinomas datasets (TCGA_BRCA_GSNP6raw-2015-02-24 and TCGA_BRCA_exp_HiSeqV2-2015-02-24) using the Cancer Genome Browser (https://genome-cancer.ucsc.edu). The YAP/TAZ signature (genes bound and regulated by YAP/TAZ in breast cancer cell lines) was taken from Zanconato et al. (2015). The Myc gene signature was taken from Ji et al. (2011). The statistical track (Fig. 4E, lower panel) shows the logarithmic plot of p-values for each genomic position/gene, where the center line indicates a p-value of 1. The direction of the test is shown by whether the bar is above or below the center line. In the case of a t-test, a bar above the line indicates that the red subgroup (basal-like tumors) is greater than the green subgroup (lumal/Her+); a bar below the line indicates that the green subgroup is greater than the red subgroup. The height of the bar (either above or below the center line) is proportional to the significance of the corrected p-value (-log p; FDR: Bonferroni corrected Student's T-test); the higher the bar, the lower the p-value, and the stronger statistical difference between the two subgroups. The bar is colored either red or green if the p-value is less than 0.05.

Statistical analysis. All the experiments were performed on biological replicates unless otherwise specified. Sample size is reported in the figure legends. Two-tailed Student's t-test was used to calculate P values; significant values are specified in the figure legends.

References

Bianchi V, Ceol A, Ogier AG, de Pretis S, Galeota E, Kishore K, Bora P, Croci O, Campaner S, Amati B et al. 2016. Integrated Systems for NGS Data Management and Analysis: Open Issues and Available Solutions. *Front Genet* **7**: 75.

Bindea G, Mlecnik B, Hackl H, Charoentong P, Tosolini M, Kirilovsky A, Fridman WH, Pages F, Trajanoski Z, Galon J. 2009. ClueGO: a Cytoscape plug-in to decipher functionally grouped gene ontology and pathway annotation networks. *Bioinformatics* **25**: 1091-1093.

Campaner S, Doni M, Hydbring P, Verrecchia A, Bianchi L, Sardella D, Schleker T, Perna D, Tronnersjo S, Murga M et al. 2010. Cdk2 suppresses cellular senescence induced by the c-myc oncogene. *Nat Cell Biol* **12**: 54-59; sup pp 51-14.

Kishore K, de Pretis S, Lister R, Morelli MJ, Bianchi V, Amati B, Ecker JR, Pelizzola M. 2015. methylPipe and compEpiTools: a suite of R packages for the integrative analysis of epigenomics data. *BMC Bioinformatics* **16**: 313.

Li H, Durbin R. 2009. Fast and accurate short read alignment with Burrows-Wheeler transform. *Bioinformatics* **25**: 1754-1760.

Love MI, Huber W, Anders S. 2014. Moderated estimation of fold change and dispersion for RNA-seq data with DESeq2. *Genome Biol* **15**: 550.

Sabo A, Kress TR, Pelizzola M, de Pretis S, Gorski MM, Tesi A, Morelli MJ, Bora P, Doni M, Verrecchia A et al. 2014. Selective transcriptional regulation by Myc in cellular growth control and lymphomagenesis. *Nature* **511**: 488-492.

Schneider CA, Rasband WS, Eliceiri KW. 2012. NIH Image to ImageJ: 25 years of image analysis. *Nat Methods* **9**: 671-675.

Shannon P, Markiel A, Ozier O, Baliga NS, Wang JT, Ramage D, Amin N, Schwikowski B, Ideker T. 2003. Cytoscape: a software environment for integrated models of biomolecular interaction networks. *Genome Res* **13**: 2498-2504.

Subramanian A, Tamayo P, Mootha VK, Mukherjee S, Ebert BL, Gillette MA, Paulovich A, Pomeroy SL, Golub TR, Lander ES et al. 2005. Gene set enrichment analysis: a knowledge-based approach for interpreting genome-wide expression profiles. *Proc Natl Acad Sci U S A* **102**: 15545-15550.

van Riggelen J, Muller J, Otto T, Beuger V, Yetil A, Choi PS, Kosan C, Moroy T, Felsher DW, Eilers M. 2010. The interaction between Myc and Miz1 is required to antagonize TGFbeta-dependent autocrine signaling during lymphoma formation and maintenance. *Genes Dev* **24**: 1281-1294.

Zhang Y, Liu T, Meyer CA, Eeckhoute J, Johnson DS, Bernstein BE, Nusbaum C, Myers RM, Brown M, Li W et al. 2008. Model-based analysis of ChIP-Seq (MACS). *Genome Biol* **9**: R137.
